# Returning to Work after the COVID-19 Pandemic Earthquake: A Systematic Review

**DOI:** 10.3390/ijerph19084538

**Published:** 2022-04-09

**Authors:** Elpidio Maria Garzillo, Arcangelo Cioffi, Angela Carta, Maria Grazia Lourdes Monaco

**Affiliations:** 1 Department of Prevention, Abruzzo Local Health Unit No. 1, 67100 L’Aquila, Italy; egarzillo@asl1abruzzo.it; 2 Section of Occupational Medicine, Department of Diagnostics and Public Health, University of Verona, 37134 Verona, Italy; arcangelo.cioffi@univr.it (A.C.); angela.carta@univr.it (A.C.); 3 Occupational Medicine Unit, University Hospital of Verona, 37134 Verona, Italy

**Keywords:** SARS-CoV-2, COVID-19, return to work, back to work, fitness for work, teleworking, psychosocial stress, mental health, healthcare, workplace

## Abstract

Background: The ongoing SARS-CoV-2 pandemic has disrupted life and work habits and has produced landmark changes worldwide. This systematic review aimed to analyse the management of Return to Work (RTW) by work organisations following the virus spread. Methods: A selection of 2477 papers, using string research on PubMed, Embase, Web of Science and Scopus from January 2020 to October 2021, were analysed. Results: Fifty-one articles were finally included, and the results obtained were discussed from three different points of view. Twenty articles concerning ‘Remodelling of Work Organization’ proposed some model strategies for resumption to work. Twenty-one papers, including ‘Clinical Evaluation of Workers’, mostly explored the psychosocial impact of returned workers. Finally, twelve articles explored the best ‘Testing Strategies related to RTW’. Despite the heterogeneity of included articles, several interesting approaches have emerged in managing RTW. Conclusions: The reported experiences could help to develop an RTW model for COVID-19 and future pandemics.

## 1. Introduction

The ongoing SARS-CoV-2 pandemic still represents a public health emergency that has affected daily social life, including the workplace, worldwide. Since the declaration of COVID-19 as a pandemic by the World Health Organization (WHO) on 11 March 2020 until 14 March 2022, more than 460 million confirmed COVID-19 cases, including more than 6 million deaths, have been reported [1]. The diffusion of numerous variants influenced the virus’s spread by forming several waves, unsynchronised across the world [2]. Lawmakers in several countries quickly produced many different regulations to counter and contain the viral spread to help the struggling occupational settings, involving restrictions and forced closure of work activities. The first rapid but equally invasive containment measure was undoubtedly the lockdown. This measure reduced COVID-19 mortality relative to the pre-vaccination era, but it affected mental and physical health, significantly impacting the economy [3]. Italy also adopted lockdown as its first containment measure, later introducing less stringent measures to modulate the effects on occupational and public health and the economy [4]. Consequently, different operating protocols and regional guidelines addressed at companies were published, with some differences between healthcare and non-healthcare environments. Numerous measures adopted by companies allow maximum protection of workers, representing help to control the spread of clusters within the production areas. In this context, resumption to work for both past-infected and healthy workers represents a primary management challenge [5,6,7].

The epidemiological data analysis provided direct feedback on the effectiveness of containment measures since the curve of the total COVID-19 cases shows a graphic flattening and a reduction in new infections. The restart in the most critical job sectors considers the very delicate phase of activity resumption for workers worldwide by adopting protocols aimed at performing work in the safest possible way. In this pandemic, Occupational Physicians (OPs) are playing a key role in monitoring the workers’ health, operating to avoid the spread of pandemic clusters at workplaces, and developing practical guidelines for the Return to Work (RTW) [8].

RTW impacts the quality of life of every worker, especially in the early pandemic stage, where no additional prophylactic measures such as vaccines were available. A critical aspect is supporting workers’ health and understanding their stress. Indeed, occupational risk exposure to COVID-19 has increased the work burden after RTW and has been associated with long-term depression, anxiety, and insomnia [9].

Starting from the updated literature, some studies developed frameworks or models adapted to specific occupational scenarios (e.g., Susceptible–Exposed–Infectious–Removed model—SEIR—remote working, systems based on big data and artificial intelligence). These approaches aimed to guide the risk assessment, simulating exposure scenarios to orient the safest RTW policy for each occupational sector with different job demands, such as manufacturing, trade, education or other services.

The healthcare setting is undoubtedly the most involved working area, representing a point of maximum exposure for health workers (HWs). In this context, RTW criteria had been proposed by national scientific organisations and several medical associations editing by specific professional needs, such as dermatologists, vestibular clinicians, cardiologists, otorhinolaryngologists, spine surgeons, general surgeons, paediatric dentists, nephrologists, sports medicine staff, and pulmonary and sleep medicine services [10,11,12,13,14,15,16,17,18,19,20,21]. Outside of healthcare settings, RTW proposal criteria have been developed for offshore workers, sex workers, and military personnel [22,23,24]. The building and construction sectors seem to have been extensively studied; Falorca JF et al. analysed the working organisation of building sectors during the pandemic, proposing a model for an effective, sustainable, and framed workers’ daily management concerning the COVID-19 emergency [25]. Regardless of the occupational setting, the risk assessment, and more specifically the evaluation of each working area, plays a pivotal role in all the organisational processes in the pandemic era [26]. Carvalhais et al. highlighted the need to reassess all occupational risks, particularly biological ones, for all workplaces. An optimal risk assessment should be based on specific working conditions, interpolating all measures already implemented with any possible adjustments [27].

Other containment measures are represented by contact tracing and people testing, also combined. The most sensitive test for SARS-CoV-2 detection is real-time polymerase chain reaction (Rt-PCR), even if positive results may also be due to an inactive virus or residual viral nucleic acid in noninfectious conditions [28]. These aspects could create many difficulties, especially in the workers’ RTW management, due to a scientific disagreement on the duration of the viral contagiousness and infectiveness, almost related to periodic SARS-CoV-2 variant diffusion [29]. Some research has questioned the true meaning of the Rt-PCR long-term positivity, the implications for practice guidelines and their correlation with the RTW process [30,31,32,33]. Perspectives on the usefulness of seroprevalence in the early stages of the pandemic have also been explored by researchers [34,35,36]. Nevertheless, SARS-CoV-2 IgM-IgG antibody testing showed limitations in documenting early-stage and late-stage infection due to low sensitivity in making diagnoses. However, serological assays can be used to support nucleic acid amplification tests.

Furthermore, the OPs must consider workers’ clinical conditions and susceptibility to SARS-CoV-2 risk exposure for Fitness for Work assessment in the RTW stage [37,38]. Therefore, the management of work disability in the pandemic and post-pandemic era, including workers infected by SARS-CoV-2, requires an interdisciplinary approach, helping with future RTW planning [39].

This research aims to systematically review the current scientific literature to summarise the strategies concerning returning to work in this delicate pandemic stage, with particular regard to:

- Remodelling of work organisation;
- Clinical evaluation of workers;
- Testing strategies related to RTW.

## 2. Methods

### 2.1. Data Sources and Searches

According to the PRISMA (Preferred Reporting Items for Systematic Reviews and Meta-Analyses), a systematic review with meta-analysis was conducted [40]. Articles published between 1 January 2020 and 29 October 2021 were searched through PubMed, Embase, Web of Science and Scopus databases, using combinations of the terms [(“COVID-19” OR “SARS-CoV-2”) AND (“Return-to-Work” OR “Back-to-Work”)]. Additional studies were taken from the reference lists of previous review articles, and citations of relevant original articles were screened.

### 2.2. Study Outcomes and Selection

**Figure 1:**
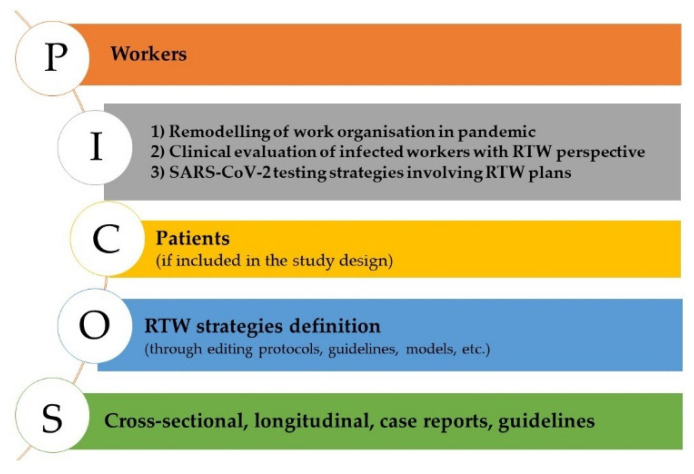
PICOS strategy adopted for studies selection. P = Population; I = Intervention; C = Control; O = Outcome; S = Studies; RTW = Return to Work.

The inclusion criteria were defined according to the PICOS strategy (Figure 1) [41]. Regarding the population (P), articles focused on workers’ or workplaces’ interventions were included only. Thus, articles exclusively relating to public health intervention were excluded. For intervention (I), based on the complete picture of the selected papers, the authors identified three points of view that could represent a practical issue for all interested stakeholders. The first theme is the ‘Remodelling of Work Organisation’, which includes all the workplace interventions facing the pandemic emergency (i.e., remote working). ‘Clinical Evaluation of Workers’ is another aspect that influences the resumption to work. Finally, an issue that polarised much occupational intervention during the pandemic was ‘Testing Strategies related to RTW’. Considering workers as the population, patients (if included in the study) were considered controls (C). The study’s core is RTW and ‘RTW strategies definition’ represented the primary outcome (O), including guidelines, on-the-field experiences, and theoretical models. Given the topic’s novelty, the authors held that even works with small cases could contribute to the purpose of this research; thus, case reports, as well as cross-sectional and longitudinal guidelines, were considered as studies (S) included.

Articles in English and Italian only were included. After removing duplicates, the following study types were excluded: review, editorials, letters to the editor, and comments.

Two reviewers independently screened the citations (title and abstract) identified from all sources. Subsequently, full-text articles were reviewed to determine the final set of eligible studies. Disagreements were resolved by discussion with the remaining authors. The selection process was carried out using some freeware (Zotero, Rayyan) [42,43].

### 2.3. Data Extraction, Synthesis, and Analysis

**Figure 2:**
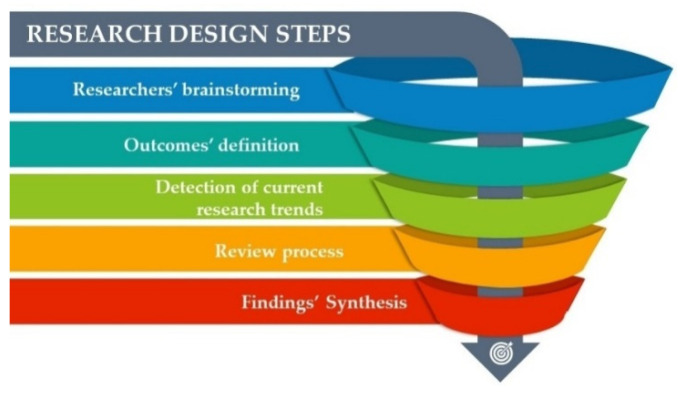
Overall research design steps.

A data extraction form was developed to determine which variables to extract using Microsoft Excel [44] The following items were included: article identifiers (authors, year of publication, country); study identifiers (setting, sample size, design); the aim of the study; and main results. According to its procedure and strategy phase, the overall research design is schematised in Figure 2.

Some papers that did not match the inclusion criteria were also commented on if relevant to improving issue analysis in the Discussion section.

In addition, a knowledge map was constructed using VOSviewer version 1.6.18, a software tool for building [45], to calculate a co-occurring relationship among keywords (co-word analysis).

## 3. Results

**Figure 3:**
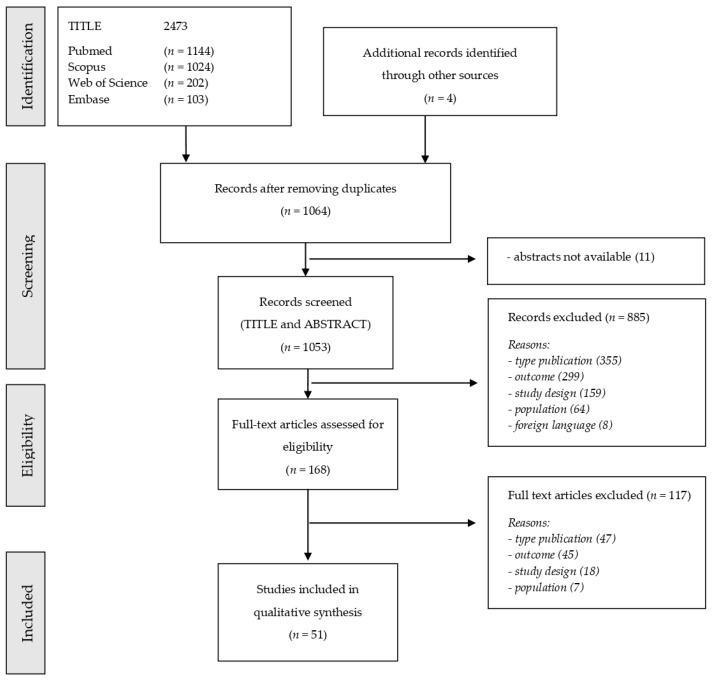
Flowchart diagram depicting the different phases of the systematic review following the Preferred Reporting Items for Systematic Reviews and Meta-Analyses (PRISMA) statement.

A total of 2477 papers were identified, and after removing duplicates, 1053 available articles were selected. According to the inclusion and exclusion criteria, about 95% of the selected papers were excluded, and 51 articles were included in the final analysis (Figure 3) [46,47,48,49,50,51,52,53,54,55,56,57,58,59,60,61,62,63,64,65,66,67,68,69,70,71,72,73,74,75,76,77,78,79,80,81,82,83,84,85,86,87,88,89,90,91,92,93,94,95,96]. The selected articles were then grouped according to three outcome approaches as follows: ‘Remodelling of Work Organisation’ (19 articles), ‘Clinical Evaluation of Workers’ (20 articles), and ‘Testing Strategies related to RTW’ (12 articles). The collected study types were as follows: cross-sectional study (33), cohort study (10), modelling study (5), case model study (1), and guidelines (2). The results are summarised in Table 1, Table 2 and Table 3.

**Figure 4:**
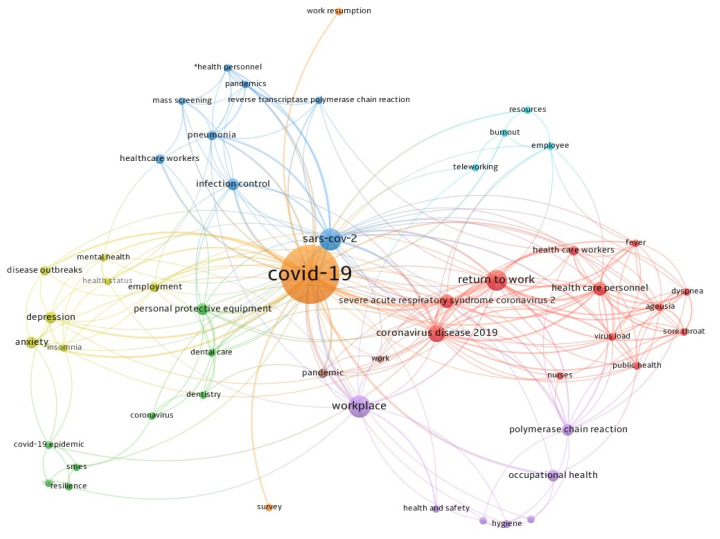
The co-words analysis of 51 studies included. The size of each node indicates the occurrence of the keyword in all 51 publications. The thickness of each link indicates the strength of the co-occurrence relationship between two keywords. The distance between two nodes indicates the relatedness of their links.

In the co-word analysis, the minimum number of occurrences of a keyword was 2, and of the 438 keywords, 70 keywords met the threshold. Among these, 51 keywords were considered relevant. Based on this bibliometric analysis, it was noted that several symptoms associated with COVID-19 were investigated, i.e., ageusia, sore throat, fever, and dyspnoea, in addition to mental health status impairment (depression, anxiety, insomnia, burnout). Explored workplaces included healthcare, dental staff, and small and medium enterprises (SMEs). Figure 4 shows the network visualisation of the keywords.

## 4. Discussion

The ongoing pandemic has produced a profound reshaping of all occupational health and safety (OHS) activities, from fitness for work to RTW, moving the entire world labour organisation from its foundations and constituting a starting point for reflection on the real need for some attitudes (i.e., remote working vs. in-person working). Therefore, in our opinion, the era we are living in and that we will live in post-pandemic is better represented by the metaphor of an earthquake rather than by other metaphors (tornado, tsunami), which describe the epidemiological phase wave but do not reflect the profound changes that SARS-CoV-2 had on occupation worldwide.

The evaluation of the articles included in this systematic review considered the evolution of the literature knowledge relating to the rapid development of new types of diagnostic tests, the onset of brand-new SARS-CoV-2 variants and the introduction of the vaccine prophylaxis.

The selected articles were grouped and commented according to three outcome approaches: ‘Remodelling of Work Organization’, ‘Clinical Evaluation of Workers’, and ‘Testing Strategies related to RTW’.

### 4.1. Remodelling of Work Organisation

The pandemic emergency, and the measures to counter it, represent the primary work activity of all the personnel involved in the organisation of work processes. The absence of vaccine prophylaxis that characterised the first pandemic wave forced all working systems to identify effective strategies to limit the SARS-CoV-2 spread at the workplace. The measures to contrast the risk from SARS-CoV-2 have been subject to constant re-evaluation as to their real effectiveness, and the acquired experiences have been published in the scientific literature. The first actions taken by all stakeholders were to quickly assess the additional risk from the virus diffusion and exclude vulnerable workers from potential exposure to SARS-CoV-2 in the workplace, also involving the OPs in these actions. The management should understand the importance of a participated approach in promoting and educating health behaviour and update the latest pandemic knowledge to identify whether changes or additional measures need to be taken [56]. Several strategies mitigate SARS-CoV-2 occupational outbreaks and promote remodelling working activities.

#### 4.1.1. Models for RTW Management

Several models for a safe RTW have been proposed, often based on algorithms developed by available data or predictive analysis. The susceptible–exposed–infectious–removed (SEIR) model to simulate SARS-CoV-2 outbreaks was one of the most used to adopt a safe strategy for work resumption.

Ge et al. showed China’s progress in business resumption strategies, summarising them into five categories for exploring the SARS-CoV-2 transmission: direct-based resumption; risk-based resumption; order-based resumption; theme-based resumption; hierarchy-based resumption [50]. The authors’ simulations showed that China’s business resumption model (except direct-based resumption) can still reduce COVID-19 prevalence with the current control measures. The authors also highlighted that a business resumption strategy, including remodelling the work organisation, could significantly influence COVID-19 prevalence reduction.

Zhao et al. elaborated a dynamic model integrating SARS-CoV-2 protection and control policies during the RTW phase with the SEIR model [64]. Different simulations suggested that the combination of quarantined and staged work resumption approaches was the most conservative and safest policy from a virus control perspective.

Zhang Q. et al. reported a case model study in which the measures for the RTW phase involved work resumption preparation, facilities and employee activity management [63]. Above all, the authors have also shown that these measures could be influenced by some ‘preconditions’, such as social culture (i.e., the privacy standards and freedom consideration), national/local OHS regulation and temporary guidelines, and finally, the OHS practices at the company level.

Among the proposed safety frameworks, Brosseau LM et al. adapted a ‘control banding model for aerosol-transmissible infectious disease’, identifying source and pathway controls to reduce the need for facial masks or other barriers. In the same paper, these authors have proposed this model in specific contexts, such as healthcare (ophthalmologists), transport workers, warehouse workers and police patrol officers [47].

Small and medium-sized enterprises (SMEs) in developing countries may experience more difficulties adopting strategies and protocols to mitigate COVID-19, resulting in a delayed RTW phase. Robinson J et al., for example, highlighted limitations in recalling the employees back to work in countries where clear guidelines to safely RTW or a national OHS system is not available [57]. RTW is a delicate phase because if work resumption causes a ‘secondary infection’ phase, it might also cause a ‘secondary shutdown’ phase [63].

Other built models are based on predictive analysis based on an algorithm, such as a regression equation. Tkatek et al. proposed a sophisticated system using big data and artificial intelligence to ensure return to work in the fastest and safest way, while those who could not work safely continue to stay at home [62]. Li Z. et al. proposed an innovative safeguard system consisting of two phases of screening, matching tests and symptom monitoring to provide adequate protection during the working period and prevent cluster infection [97].

Lichtman et al. developed an electronic, smartphone-compatible survey tool using Qualtrics software to rapidly identify and address presenteeism during COVID-19. A daily symptom monitoring tool could be a flexible method to avoid symptomatic HWs coming back to work [52].

#### 4.1.2. Remodelling in Healthcare Settings

Job remodelling has affected the whole working environment, but the most substantial changes have been registered in the Healthcare Sector, involving the RTW management. Soneru CN et al. highlighted that RTW guidelines for infection management varied by healthcare facility in different countries regarding symptom resolution, number of days after symptoms started, and negative serial testing for SARS-CoV-2. Regarding the impact of the virus spread on paediatric anaesthesia staff, these authors also showed that some workers preferred to avoid treating SARS-CoV-2-positive cases based on age, comorbidities, and pregnancy status of the paediatric anaesthesia staff [59]. Calderwood MS et al. reported that variation in the type of isolation precautions used for specific procedures during the COVID-19 pandemic was higher than expected among healthcare facilities dealing with other viral respiratory pathogens. Moreover, they reported uneven adherence to the USA Center for Disease Control non-test-based return-to-work criteria among the investigated healthcare facilities [48].

Dental operators seem to be one of the most involved working settings, probably due to the production of aerosols during most of the medical activities performed on patients. Consequently, several types of research on the containment of SARS-CoV-2, the remodelling and consequential RTW for this particular working sector have been published. Expósito-Delgado et al. showed that dental hygienists faced the RTW with different strategies aimed at infection control and ensuring the safety of all involved professionals. They also highlighted that the remodelling of working tasks should have been carried out progressively while limiting the production of aerosols [49].

According to the literature data, perceived risk influences RTW, especially in high-risk exposure areas, such as dental staff, and is also influenced by high-risk exposure areas. Salgarello S. et al. reported a different geographical resumption within a unique nation (Italy), probably due to the further evolution of the virus over time; the authors highlighted the necessity of dentists to modify the adopted recommended precautionary guidelines according to the specific dental treatments [58].

Re-deployment to deal with pandemic emergencies is another aspect related to the remodelling of the work organisation. Health services need to make a systematic and comprehensive plan for the RTW stage, also including the experience of reassigned medical and other staff trainees. No studies matching the review’s inclusion criteria were found.

Implementing a unique RTW policy for healthcare professionals is required, at least within those nations providing centralised health governance. Onesti CE et al. reported differences in requiring an Rt-PCR negative test for RTW in some oncological care organisations. These authors vocalised that the relative efficacy of many pragmatic interventions needs to be further analysed in extensive observational studies [55].

#### 4.1.3. Teleworking

Teleworking, or remote working, as a resumption of normal activities, was also considered an RTW chance. This working model should be used during this period as a control measure against the virus spread, but at the same time, its consequences on the workers’ private life need to be studied.

A comprehensive approach to developing a successful RTW policy was addressed by Taylor TK et al. in the published guidelines promoted by ACOEM [60]. These authors stressed the importance of developing procedures for dealing with infected and exposed employees in the workplace, analysing how to harmonise this phase with, for example, the viral shedding phenomenon. The same authors also emphasised the importance of the risk analysis approach in commuting and the SARS-CoV-2 exposure grading. Further, in another publication, Taylor TK et al. underline the importance of teleworking in the work re-organization to maintain employee mental health [61]. They also proposed practical RTW considerations regarding occupational activities such as the food industry, general office settings/warehouses, retail, healthcare, long-term care facilities, transportation and travel, construction, and marine and offshore sectors.

Remote working has blurred the lines between work and personal lives, negatively impacting spare time or converting a place for rest into a workplace. Niu Q et al. reported that workers showed more physical symptoms performing teleworking, which may be related to an unprofessional office environment and more work–family conflict [98]. Wood JS et al. analysed the potential problem affecting the well-being of teleworkers in a UK university (such as work–home interface, homeworking and COVID-19-specific factors). The ongoing pandemic has contributed to short-term fluctuations in the health of these employees working at home [99].

Hybrid working, a combination of remote working with a flexible regime, could be an intelligent resource in the next post-pandemic era. Radonić et al. reported some practical implications to be used by decision-makers in managing the hybrid workplace models, especially in re-thinking the working setting by reshaping the post-COVID-19 working environments [100].

However, according to the review’s inclusion criteria, these last articles were excluded from the review process [98,99,100].

In line with teleworking, remote medical assistance, also called *telemedicine* or *telehealth*, has been implemented. Telemedicine (“healing at a distance”) signifies the use of information and communication technologies to improve patient outcomes by increasing access to care and medical information. To date, World Health Organization (WHO) defines telemedicine as “*The delivery of health care services, where distance is a critical factor, by all health care professionals using information and communication technologies for the exchange of valid information for diagnosis, treatment and prevention of disease and injuries, research and evaluation, and for the continuing education of health care providers, all in the interests of advancing the health of individuals and their communities*”. Telemedicine and telehealth are used interchangeably in this research, even if there are distinctions between the two terms in the scientific literature [101].

Considering the use of telehealth as an outcome for injured workers themselves (needing rehab after work-related injuries), Gross DP et al. showed that workers assessed using remote assistance were significantly less likely to be judged as ready to return to pre-accident functional work levels and more likely to be recommended modified work duties [51]. The use of the telehealth approach would therefore lengthen recovery times and the RTW phase for injured workers.

Another RTW critical issue is fitness for teleworking, establishing if the worker is not susceptible to increased work-related stress, poor performance or burnout if remote working is allowed. Barriga Medina HR et al. reported high levels of work–family conflict, especially in those who teleworked more than eight hours per day, manifested primarily as greater exhaustion. Nevertheless, according to these researchers, teleworking overload did not affect the work–family conflict and burnout relationship [46].

Marzban S et al. reported that, after this pandemic earthquake, workers preferred to have a better supply of flexible working arrangements, perhaps alternating remote and in-person working 2–3 days a week [54]. In the same paper, these authors also showed that employee and organisation perspectives on the ‘new normality’ after the COVID-19 pandemic are nearly aligned, believing that adaptability and flexibility will be the likely post-pandemic era. In this work, a different approach was also highlighted about the working generation: the younger ones (Generation Y or millennials) would have suffered more from the isolation related to remote work. At the same time, some workers would like to return to the office with caution, highlighting the importance of social connection and the separation between work and life.

In healthcare settings, remote working strategies directly affect healthcare performance, too. Ashry AH asked enrolled doctors to grade their satisfaction about application quality to perform a satisfying remote examination and expand the application of telemedicine visits after the pandemic. Most enrolled physicians preferred to develop the application of telemedicine experience after the pandemic [102].

Considering that the pandemic affected the entire world in alternating phases, it is impossible to summarise and compare all the different management approaches of the countries involved. In addition, it must be taken into account that each country, even before the pandemic, had different approaches to work organisation and occupational health and safety (including general pandemic strategies). However, according to this study’s outcome and inclusion/exclusion criteria, the paper cannot report all differences in national/regional regulations worldwide. For example, in Italy, as of late January 2020, the government has promulgated numerous regulations and guidelines aimed at containing the virus spread and managing RTW. Still, following the selection criteria, these documents were not included in the study.

In conclusion, the remodelling of working activities in the pandemic and post-pandemic period should consider the re-assessment of the workplace’s risk exposure and the following adoption of technical measures for virus spreading containment. The evaluation of alternative working modalities, such as teleworking, needs to be studied by enterprises management. The emerging risks related to these different working modalities should be considered ergonomic and psychological risks. Furthermore, all the management must include customised decisions for each worker, with the OPs support to evaluate the worker’s clinical condition in the Fitness for Work process.

### 4.2. Evaluation of Clinical Condition, Health Surveillance and RTW

The evaluation of workers’ clinical conditions and Fitness for Work are strictly related. OPs play a key role in providing helpful information for an integrated approach to RTW management. In this pandemic era, all the medical evaluations performed by OPs, including the health surveillance programs, underwent profound reassessment due to the virus spread. Implementing these programs has successfully reduced the frequency and mortality of SARS-CoV-2, especially among HWs [103,104]. The analysis of clinical course and the time from COVID-19 symptom onset, or SARS-CoV-2 test positivity, to come back to the workplace is a parameter for managing solutions by OPs. Ganz-Lord FA et al. highlighted that the median time from symptom onset to RTW for non-hospitalised HWs was 15 days. Shortness of breath, fever, sore throat, and diarrhoea were significantly associated with longer durations from symptom onset to the resumption of normal working activities [67].

The health workforce in each country has been complexly tested to allow all available and suitable personnel to return to fieldwork. For this reason, in this ‘forced / emergency RTW process’, it was necessary to evaluate the available workforce by OPs also based on the physical conditions of the active retired population, in which, inevitably, several pathologies due to ageing or disabilities have been found. The available literature concerning this issue is lacking. Ly DP reported that about 15% of the HWs who returned to the labour force were affected by a disability or had older parents at home to take care of; all these issues may represent challenges and limits for back to work [73].

The psychosocial effects of the pandemic is one of the most investigated topics, especially in China, with a strong impact on the RTW phase.

#### 4.2.1. Psychosocial Aspects

Since the first SARS-CoV-2 wave, researchers have investigated the relationship between psychosocial stress caused by the pandemic and job stress. In the last two years, people experienced social isolation, lifestyle disruption and loss of personal income due to prolonged lockdown and business closure. In this context, Du Y et al. stated that job burnout, as a hostile working state linked to the continuous and intense working environment, negatively affects employees’ safety behaviour. For this reason, enterprises should reduce the job burnout risk caused by the pandemic situation at work resumption. Moreover, the authors suggest improving the employees’ perceived insider status, as the value that employees feel and the degree to which they are treated as “insiders” by the organisation to avoid the harm of COVID-19 [65].

Yang Q et al. investigated the psychosocial stress in returned workers, exploring the solution strategies and the role of the perceived organisational and social support. According to other literature evidence, these supports seemed to weaken the impact of psychosocial stress on the RTW related stress. The organisational management should play a leading role in reducing the synergy impact between psychosocial stress related to the pandemic emergency and the routinely working stress linked to the job tasks. The authors also evaluated that the workers’ stress response to COVID-19 depended on their pandemic awareness, highlighting an inverse correlation between accurate pandemic employees’ awareness and psychosocial stress impact on work stress [81].

These anxious synergy conditions affect transversally all work contexts, not least the sports fields. A study conducted on professional footballers has shown that COVID-19 stress may be added to performance anxiety, typical of such contexts, setting the stage for a negative impact on the professionals’ performance [74].

Tan W et al. found that 10.8% of the workforce met the diagnostic criteria for Post-Traumatic Stress Disorder (PTSD) when returning to work during the pandemic, suggesting that the RTW experience did not increase the prevalence of psychiatric symptoms such as PTSD, depression, anxiety and stress when compared to results about the COVID-19 outbreak literature data. These findings are probably due to the confidence instilled by prevention measures, such as hand hygiene, wearing face masks, and organisational measures, including significant improvement of workplace hygiene [78]. On the contrary, some evidence (not included in this systematic review) suggests that the COVID-19 pandemic added an extra burden on the routine job demands, especially in those working areas exposed to high risks, such as dental health professionals, with a significant disruption of healthy work–life balance, sleeping quality, and stress load [105]. These findings are consistent with Liu Z et al., who reported that enrolled employees expressed limited willingness to return to the workplace, expressing a preference for not returning yet or continuing homeworking. Indeed, these results reveal a phenomenon of reluctant returning when people may feel forced to return to workspaces, particularly women, non-Caucasians, and employees living in multi-generational households [72].

Several studies focused on what employees can do to facilitate the RTW phase and how job remodelling could protect from SARS-CoV-2 exposure, including the psychological effects on workplace-related virus fear.

Yuan Z et al. [82] reported that work reattachment about resuming regular job activities was related to higher levels of job engagement, lower levels of work withdrawal and higher levels of personal protective equipment use. Moreover, work organisation managers could help employees regain focus in this ongoing pandemic. In this context, the role of the OPs seems crucial in expressing Fitness for Work weighted for a post-pandemic brand new normality.

HWs’ psychosocial risks associated with the management of the pandemic emergency is another peculiar topic in the scientific literature, potentially related to the higher risk exposure to SARS-CoV-2. Actions aimed at correctly assessing and managing the psychosocial risks linked to the ongoing emergency must always be considered. In the healthcare setting, management must consider the coping strategies for HWs work resumption to recover the workers’ enthusiasm to face future uncertainties through addressing the threats that workers bring to their workplaces, such as social hypochondria, suspicion, and massive distrust; promoting diversity and innovation; recovering trust and inspiration [106]. As mindfulness, some coping strategies should help reduce RTW-related stress and anxiety, increasing resilience and improving flexibility [107]. Zheng N et al. analysed these factors, showing that managers should adopt effective intervention measures to address returning personnel’s physical and mental health by considering several individual parameters, such as roles and cultural adaptation, self-efficacy, optimism, and resilience [84]. Another study described modification status of nursing performance in some previously infected HWs after work resumption, such as declined ethical values or *coronophobia*, or the fear of re-infection [75]. Rex DK et al. have discussed concerns of endoscopy staff when resuming elective endoscopy; even after instituting new safety and protective measures, 35% of them remained very or somewhat concerned [76]. Much evidence has shown a high incidence of burn-out, especially in frontline HWs, often worried that transmission and relative exposure to risk were not clear yet. The uncertainty derived from the lack of knowledge about the virus and its occupational management generated anxiety, depression, and insomnia during the first phase of RTW [80]. For all these reasons, HWs need to be updated about the knowledge and awareness of SARS-CoV-2 by promoting appropriate and continuous training to increase HWs’ consciousness and to be integrated into clinical management, attitude and practices while managing COVID-19 cases. The use of updated information regarding SARS-CoV-2 may improve mental health in the workplace. Retraining based on the latest available pandemic evidence should always be proposed to workers and help to cope with the anxiety and stress of work resumption [108]. A participated training process plays a key role in COVID-19 prevention and safe RTW. These can also be included as preventive strategies and preparedness plans [109]. Lai R et al. highlighted that hospital management should strengthen the training related to the pandemic issue through intervention and online or offline education [70].

The resumption of working activities exposes all workers to different concerns regarding workplace reopening during the pandemic. According to Griffiths D et al., problems were more prevalent for workers reporting psychological distress and financial stress and for those exclusively working from home. Concerns regarding work and home life changes were more common for female workers and partners with dependent children. Regarding infection risk, problems are common for HWs and the retail, accommodation, and food service industries [71].

RTW is a critical point involving psychosocial aspects. This phase represents, for some workers, the hope to come back to normality, restarting the routine activities performed in the pre-COVID-19 era. Many workers had to interface with a very different normality, characterised by a substantial modification of all the activities and habits (wearing face masks, social distancing at work, frequent COVID-19 testing, limited public transport, etc.). For this reason, RTW must be adequately assessed and managed, potentially constituting a coping strategy for anxiety and depression generated by the pandemic condition.

#### 4.2.2. Long COVID

Long COVID, or post-COVID syndrome, is a newly emerging health problem consisting of a disease complication linked to the SARS-CoV-2 infection, still in the nosological definition phase. The WHO has proposed the moniker ‘post-COVID-19 condition’, as persistent symptoms that usually occur three months from the onset in individuals with past confirmed or probable SARS-CoV-2 infection and persisting for at least two months and that cannot be explained by an alternative diagnosis [110].

It may lead to chronic health problems due to different complications of COVID-19, such as long-lasting neurological, cognitive, pulmonary, and cardiac diseases. In these cases, RTW may be delayed in some workers who need rehabilitation. Most patients not affected by severe COVID-19 that requires hospitalisation, even without pre-existing clinical conditions, often develop symptoms of fatigue and perceived cognitive impairment (called ‘brain fog’), resulting in severe negative impacts on the resumption of normal working activities [111]. Furthermore, workers with neurological complications were significantly less likely to resume their previous activities quickly. Frontera J et al. showed that a patient cohort characterised by COVID-19-related neurological impairment could not resume previous activities by 6 months, impacting unmeasurable socio-economic patient conditions [66]. Vanichkachorn G et al. analysed a cohort study of 100 long-COVID patients admitted to activity rehabilitation programs. The authors observed that numerous patients have not been able to return to work; returning to their previous occupation is delayed months after their initial SARS-CoV-2 infection. Only one-third of enrolled patients returned to unrestricted work duty [79].

These conditions cause health and socio-economic effects due to the global burden of COVID-19 associated with health status, which could be measured using ‘the disability-adjusted life years (DALYs)’ proposed in the Global Burden of Disease (GBD) Study for COVID-19 [112].

#### 4.2.3. Pre-Existing Medical Conditions and Vulnerable Workers

SARS-CoV-2 has infected millions of workers, including vulnerable ones due to pre-existing medical conditions (i.e., cardiac, neurological, neoplastic, metabolic pathologies), or ongoing therapies, such as immunosuppressive therapies. For these cases, Fitness for Work evaluation required a strict collaboration between organisational managers, such as Human Resource Management, and OPs, sometimes requiring support from other medical specialists, to assess the compatibility between the workers’ clinical conditions and the risk of exposure to coronavirus. The risk (re)assessment in this pandemic post-lockdown should be targeted at vulnerable workers, such as disabled staff [113].

The key role of counselling specialists in RTW for these patients was described by Ladak K et al., who interviewed Canadian doctors and questioned them about their counselling offer to those patients in RTW. The factors influencing the counselling regarding returning to routinary working activities are co-morbidities, patient age, high-risk work and vulnerable co-inhabitants living with the worker. Furthermore, some treatments, such as corticosteroid therapy, influenced RTW decision making, while the use of other drugs, such as antirheumatic drugs, biologics, or JAK inhibitors, did not prompt recommended delayed resumption to work activities or modified duties, according to the literature evidence of no negative impact of COVID-19 outcomes of these therapies, reducing the aggressive inflammatory self-response to SARS-CoV-2 [69].

Cancer is another vulnerability for SARS-CoV-2 exposure. Workers who have cancer who cannot benefit from homeworking due to their “essential role” may be forced into temporary unemployment due to their higher sensitivity to virus exposure. In some cases, these workers have a bad financial situation, forcing them to continue frequenting their workplace, resulting in increased virus risk transmission and associated distress. Cancer survivors could have less chance of re-employment after a job loss than the general healthy population [114].

Liu X et al. investigated the relationship between COVID-19 fear and work sustainability and whether it interacts with the fear of cancer recurrence (FCR) in male cancer survivors. The study found that male cancer survivors with higher levels of FCR reported lower confidence in work sustainability and may have less confidence in remaining at work [71].

Vulnerable workers could develop more PTSD or moderate anxiety for pandemic conditions. Zanghì et al. reported clinically significant PTSD-like symptoms and moderate-to-severe anxiety in 48.6% of enrolled workers affected by the chronic neurological disease when they reattempted to work [83].

Employees with high physical and occupational distress pose some challenges for employers and, more specifically, OPs. Workers with COVID-19-related lung impairment could have difficulties using masks, resulting in no easy solutions for related Fitness for Work. Several studies explored the impact of wearing different face masks among subjects with mild pulmonary diseases, such as asthma, chronic rhinitis, and chronic obstructive pulmonary disease, during simulated work tasks, both sedentary and more active ones. According to Scheid JL et al., the relationship between the power filtration of the mask and disease severity was associated with higher discomfort or inability in wearing a mask [115]. This issue could influence the fitness to stay or return to work during the pandemic era.

Employment accommodations during COVID-19 and rehabilitation efforts to assist workers with disabilities in maintaining their careers were two main missions during COVID-19. Rumrill et al. have shown the use of specific tools or planning procedures that help workers with disabilities resume or retain their employment. Most participants reported high job mastery and job satisfaction [79].

The COVID-19 era re-marked the crucial role of Fitness for Work assessment both in post-infected and healthy workers. Much of the evidence included in this review came from European countries with a grounded culture of health surveillance, a helpful tool for job reorganisation and RTW based on workers’ clinical evaluation. Other countries, in our opinion, could have additional challenges in the RTW if the health and safety system is not well developed. The research findings showed the primary need to provide careful occupational management and psychological support to vulnerable workers facing RTW. Long COVID must be considered as a brand-new clinical condition affecting daily and work activities, thus requesting a specific clinical evaluation by OPs, also from vulnerable and disabled workers.

### 4.3. SARS-CoV-2 Testing and Return to Work

During this pandemic period, the criteria for return to normal activities, including resumption to work, were mainly based on clinical and laboratory assessments of symptom resolution. At least two consecutive negative swabs were collected within 24 h in the first wave. Indications for ending isolation or precautions for people affected by SARS-CoV-2 infection are still subject to constant revision by all international scientific committees.

Workers’ screening is a primary strategy for the containment of the virus spread in the workplace: detecting SARS-CoV-2 positive cases and understanding when a worker is to be considered no longer infectious [116]. Health surveillance by testing the workers is the most protective strategy for workplace safety and RTW management. The most accurate COVID-19 diagnosis is still through the analysis of nucleic acids, that is, the demonstration of SARS-CoV2 RNA in respiratory samples [117]. Thus, the primary screening protocols for occupational and public health contexts include Rt-PCR analysis for SARS-CoV-2 by swab testing, a gold standard for infection diagnosis. Alongside this test, other more uncomplicated and rapid methods, such as serological testing of IgM and IgG anti-SARS-CoV-2, may improve the detection performance of the molecular test for screening purposes. However, serological tests cannot replace Rt-PCR in virus detection and screening [118]. Due to data variability and the lack of reporting, the antibody titre’s protective capacity and the related cut-off remain to be defined [119]. These tests are helpful to assess the overall infection rate, including asymptomatic infections, because antibodies are usually detected only 1–3 weeks after the onset of the symptoms or when Rt-PCR assays are not available. Overall, a combination of clinical, molecular, and serological diagnostic tests is highly recommended for adequate screen sensitivity and specificity, depending on the type of sample and stage of the disease [120]. For all these reasons, some literature data were reported using the Rt-PCR test and other types of tests, in combination with clinical evaluation, for health surveillance and RTW management.

Tripathy D. et al. suggest that a point-of-care rapid antigen test may be considered for preoperative patient screening, contact tracing, and RTW policies [95]. In contrast, Guarnieri et al. suggested combining Rt-PCR on nasopharyngeal swabs and rapid serologic tests for SARS-CoV-2 should be modulated to have the most accurate and implemented surveillance system [90].

Several observational studies focused on the length of PCR test negativisation, helpful information to plan workers’ tests timing for re-admission at work also incorporated in RTW national guidelines or health laws. Shenoy ES et al. reported data related to the first pandemic wave, investigating the negativisation time from the first positive molecular test to both the first and second negative ones. The median number of days from the first positive to the first negative was 17 (range 2–38); the median number of days from first positive to second negative was 19 (range 6–37) [104]. González Martin-Moro J et al. reported a median time to negativisation of about 25 days from symptom onset [89], consistent with Cariani L et al., who showed a negativisation time of 4 weeks [85], while Gombar S et al. proposed to consider a viral shedding for 33 days following symptom onset [83]. Further variable data were reported by Domeracki S et al., highlighting a time of Rt-PCR clearance ranging from 7 to 57 days, with a median of 34.5 days. These findings suggest that the length of test negativisation could be influenced by individual factors, such as occupational exposure, age, and gender [86].

Some evidence has suggested that RTW might be based on the Rt-PCR cycle threshold (Ct) evaluation and not only on the qualitative results (negative vs. positive), adding further heterogeneity in RTW protocols. The Rt-PCR Ct, which correlates with the estimated viral load, may help RTW planning and decision making based exclusively on qualitative results [86]. In this context, it is necessary to have consolidated data on the real times of SARS-CoV-2 negativisation or at least when a positive worker can be considered non-infectious to orient the screening test timing correctly.

Another critical issue regarding RTW is the test timing. Early testing after exposure, carried out by a proactive contact tracing system, is fundamental to lower the virus spread and could allow a faster RTW, limiting the staff shortage and related work overload [87].

The individuation of subjects to be screened for SARS-CoV-2 is continuously evolving concerning the new variables that have occurred, not least the vaccines’ introduction and the diffusion of virus variants, resulting in a modification of the clinical phenotype, with an increase in asymptomatic cases [121]. Therefore, asymptomatic workers represent a critical issue concerning health surveillance, Fitness for Work and related RTW policy in this COVID-19 era. Guarnieri V et al. reported that 13.3% of asymptomatic HWs after an influenza-like illness were found positive on the day of re-admission at work, sustaining the policy of the hospital management to test all workers before re-admission at work [90] and supporting the usefulness of mass testing included in the worker’s health surveillance. Mass testing, mainly performed in the healthcare setting, as the main surveillance strategy, enabled the isolation of positive workers. Porru S et al. found a 2% SARS-CoV-2 positive rate among asymptomatic HWs [92], consistent with 3% of HWs who tested positive by Rivett L et al. in the absence of symptoms [93]; another study showed a positivity rate among asymptomatic people equal to 17.6% [85]. Despite all this evidence variability, the mass test screening programs could be an effective tool for improving risk assessment, Fitness for Work evaluation and, more broadly, virus control, especially in the peak phases of virus spread. On the other hand, other containment strategies could be chosen during lower virus spread and considering the current vaccine coverage, such as personal protective equipment use only or home isolation.

Other screening campaigns were based on more specific approaches, including algorithms to select workers to be tested. Pan SC et al. reported an experience based on “hospital-wide web-based health surveillance integrated with a risk-based management algorithm and molecular testing of asymptomatic HWs”. This integrated system enabled the identification of workers at higher infection risk, early detection of asymptomatic subjects, and avoiding virus spreading at the workplace [91].

Alongside the healthcare setting’s experience, other occupational scenarios regarding these topics were evaluated, even if not strictly oriented to RTW. Lucan et al. proposed a pilot screening for essential food production workers, defining three outcomes: detecting asymptomatic/pre-symptomatic infections before a cluster risk; identifying clusters of cases to indicate potential protection breakdowns; assessing overall workplace safety by comparing company results to community rates [122].

As the above-reported evidence states, identifying SARS-CoV-2 cases by worker testing is the main instrument of screening programs and a keystone of RTW protocols. Therefore, a combined strategy of Rt-PCR and serological testing associated with clinical evaluation is recommended for effective health surveillance. The rapid evolution of the ongoing pandemic forced flexibility of all screening programs regarding the choice of appropriate tests, people to be tested, and relative timing.

**Figure 5:**
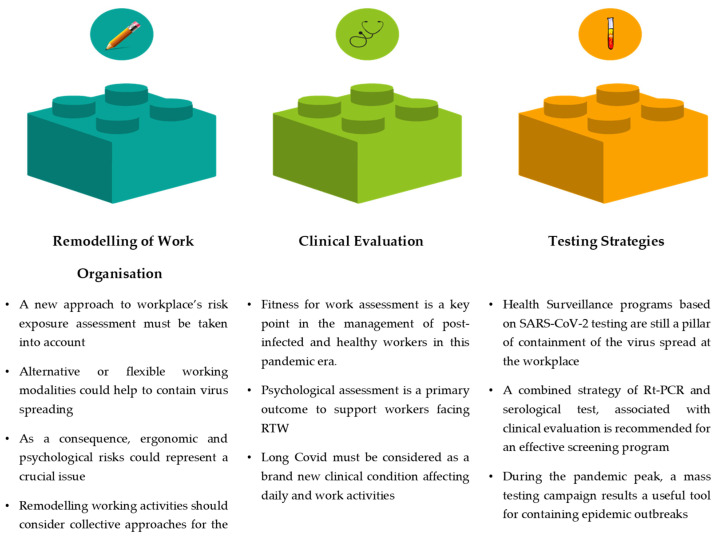
Summary of the main review findings.

The review’s key messages are summarised in Figure 5.

## 5. Strengths and limitations

This paper is one of the first systematic investigations about RTW strategies in this pandemic stage. The findings from many studies were reported in three main topics to thoroughly explore the RTW issue and provide helpful information to make a shareable model. The outcomes of this study may help us to understand the issue concerning COVID-19 effects in the workplace and may guide the development of RTW programs tailored to the needs of work settings.

This review presents some limitations. Given the novelty of the topic, papers with small samples were included in this research, representing a potential information bias. Moreover, the heterogeneity of the included articles could hinder the definition or categorisation of all studies, limiting further quantitative analysis. The limitations are due to a lack of consistency in measuring RTW: the definition of RTW was inconsistent across studies. Lastly, differences in diagnosis infection and RTW policies among countries, also linked to the pandemic evolution, most likely influenced RTW. Moreover, this continually evolving pandemic situation does not allow RTW strategies to be frozen and rigidly codified.

Despite these limitations, the authors believe that this study’s results may contribute to filling a knowledge gap about RTW management and potentially helping manage the COVID-19 impact beyond the infectious period.

## 6. Conclusions

RTW guidance changes over time in this pandemic. Although no specific tailor-made COVID-19 screening for each working reality has been developed, organizations should determine the most appropriate RTW protocol for their working setting based on occupational health professionals’ recommendations, referencing updated national/international guidance and scholarly literature, in combination with cost-effectiveness and, not least, the nature and needs of the workplace.

This study was carried out by analysing the primary international scientific databases, formulating specific queries oriented to the delicate phase of RTW, and assessing the responsiveness of occupational environments around the world regarding the organisation and the RTW management in this pandemic era. From an occupational health perspective, the results of this study might improve health and safety systems to develop management strategies not only for an endemic infection, as SARS-CoV-2 is likely to evolve, but also for use in the future pandemics related to new variants or other virus types.

## Figures and Tables

**Table 1:** Selected papers for ‘Remodelling of Work Organisation’. The table reports the characteristics and main findings of 19 articles (11 cross-sectional studies, 5 modelling studies and 2 guidelines) concerning reassessment of SARS-CoV-2 risk exposure and consequential virus spreading containment measures, in several countries such as the USA (7), China and South-East Asia (6), Europe (2), and others in different occupational settings, mainly healthcare.

Authors, Year	Country	Setting	Sample Size	Study Design	Aim of the Study	Main Findings
Barriga Medina HR et al., 2021 [46]	Ecuador	Teleworkers in Guayaquil, Ecuador.	1044 Teleworkers	Cross-sectional	To analyse the impact of work-family conflict on burnout and work overload in teleworkers during the COVID-19 pandemic.	Teleworkers are involved in high levels of work-family conflict; the impact of the work-family conflict manifested primarily in greater exhaustion; no effect of teleworking overlwork-familywork–family conflict and burnout relationship.
Brosseau LM et al., 2020 [47]	USA	Healthcare (Ophthalmologist) and Non-Healthcare settings (transportation and warehouse workers, police patrol officer)	N/A	Modelling study	To describe how a control banding model is applicable to the current COVID-19 pandemic and illustrate, using several case examples, how decisions about workplace controls for aerosol transmission are facilitated by this model and can inform the safe reopening of workplaces.	The using control banding for workers at high risk of exposure in the Return to Work (RTW) phase is unhelpful to develop effective infection and disease prevention programs.
Calderwood MS et al., 2020 [48]	USA	USA and International Healthcare Facilities	95 Healthcare Facilities	Cross-sectional	To obtain an aggregated picture of healthcare facilities’ approaches to mitigating COVID-19 transmission risk.	Variation in the isolation precautions used for specific procedures during the COVID-19 pandemic was higher than expected among healthcare facilities dealing with other viral respiratory pathogens. Moreover, the authors reported that healthcare facilities followed the Centers for Disease Control and Prevention (CDC) non-test-based return-to-work criteria for more than a half (52%)
Expósito-Delgado et al., 2021 [49]	Spain	Spanish dental hygienists	517 Dental Hygienists	Cross-sectional	To describe dental hygienists’ work status and employment patterns during RTW	86.2% followed the official recommendations to avoid contagion; 63.8% agreed with the gradual RTW by limiting the use of the aerosols; private dental hygienists identified more with RTW without restrictions (14.5%) versus those working for public service (1.2%)
Ge Y et al., 2021 [50]	China	China’s Business Enterprises	Not specified	Modelling study	To investigate the effects of different resumption strategies on COVID-19 transmission using a Susceptible–Exposed–Infectious–Removed (SEIR) model.	The hierarchy-based reopen strategy performed best when current pandemic prevention measures were maintained save for lockdown, reducing the peak number of active cases (50%) and cumulative cases (44%)
Gross DP et al., 2020 [51]	Canada	Workers’ Compensation Board of Alberta	4516 Injured Workers	Cross-sectional	To describe the transition to remote occupational rehabilitation services in response to the COVID-19 pandemic.	Workers using remote assessments were significantly less likely to be judged as ready to return to pre-accident functional work levels and more likely to be recommended modified work duties. The number of completed rehabilitation programs also reduced.
Lichtman A et al., 2021 [52]	USA	Healthcare Settings in California	5035 Health Workers (HWs)	Modelling study	To outline an effective strategy for rapid implementation of a symptom monitoring system to integrate into an adaptable model	1318 HWs had been identified as being symptomatic with testing indication. A total of 82% reported not currently staying home from work due to illness or quarantine, consistent with the high rates of ‘presenteeism’ reported in HWs.
Lu Y et al., 2020 [53]	China	Small and Medium Enterprises (SME) in Sichuan’s	4087 workers	Cross-sectional	To assess the challenges associated with work resumption and the related policy requirements.	SMEs were unable to resume work for several reasons, including a shortage of pandemic mitigation materials, the inability of employees to RTW, disrupted supply chains, and reduced market demand.
Marzban S et al., 2021 [54]	Australia	28 Australian organisations	301 employees	Cross-sectional	To describe the homeworking experience during the Australian lockdown in the middle of the COVID-19 pandemic, and to inform organisations, employees and the design of the workspaces post-2020.	Organisations reported a homeworking increase (more than 50%) and indicated employees’ productivity as the biggest concern in remote working. Employees were more concerned about their social interactions, internet connectivity, and increased workload challenges and disclosed that face-to-face interactions with their colleagues were the most important reason to return to the office.
Onesti CE et al., 2021 [55]	USA/Europe	Oncological Centres in Europe and USA	30 Oncological Units	Cross-sectional	To assess how oncology centres reacted to the health crisis related to the COVID-19 pandemic to improve oncological care and implement preventive measures.	Triage for patients with cancer was conducted before a hospital or clinic visit (90.5%), before daycare admissions (95.2%), and before overnight hospitalisation. Separated pathways for COVID-19-positive/-negative patients were organised, and permission for caregivers to attend clinic visits was limited. Telemedicine was implemented in 76.2% of the centres. RTW policies required a negative swab test in 76.2% of the centres
Pratama MR et al., 2021 [56]	Indonesia	Indonesian Companies (Healthcare excluded)	106 Participants	Cross-sectional	To assess the measures taken against the COVID-19 pandemic in workplaces.	Almost all enrolled participants have already developed the specific COVID-19 policy. A total of 91% reported a specific emergency response team and communication centre for COVID-19 at the workplace, but only 42.7% performed an emergency drill for COVID-19 cases. A total of 95.1% of the participants implemented flexible worksites and work hours to maximise the physical distancing among the workers.
Robinson J et al., 2020 [57]	Sri Lanka	Small and Medium Enterprises (SME)	14 SME operators	Cross-sectional	To explore the effects of the COVID-19 pandemic on SMEs in Sri Lanka	Some managers’ practices, such as a four-day workweek, work sharing, half-time working, etc., might help the SMEs to remain open; in addition to management local actions, Government should establish health and safety measures to ensure minimal virus diffusion at the workplace, formulating the right policies and guidelines to support the SMEs.
Salgarello S et al., 2021 [58]	Italy	Italian Dentists	1028 Dentists	Cross-sectional	To understand the procedures that were adopted in the second phase of the COVID-19 pandemic and to evaluate the dentists’ expectations and concerns about returning to normalcy	A total of 83% of Italian dentists fully restarted their activities after the lockdown, with a resumption significantly marked in northern and central Italy than in the south. Over 80% adopted the recommended precautional guidelines, modifying them according to the specific dental treatment. Additionally, 50% were confident in returning to normalcy after the COVID-19 crisis
Soneru et al., 2021 [59]	USA	Paediatric Anaesthesiology Staff Worldwide	63 Institutions	Cross-sectional	To determine how COVID-19 directly impacted paediatric anaesthesia practices during September/October 2020	N95 masks were available to anaesthesia teams at 91% of institutions. COVID-19 testing criteria of anaesthesia staff and RTW guidelines varied by institution. Structured simulation training aimed at improving COVID-19 safety and patient care at 62% of institutions. A total of 31% declared a voluntary option to not work with COVID-19-positive cases
Taylor TK et al., 2020 [60]	USA	ACOEM	N/A	Guideline	To provide RTW guidance for employers and the OPs supporting businesses in implementing safe strategies—Part I: General Guidance for Employers.	Transition phase analysis: RTW policies proposal. Employers will need to facilitate the safe return of employees through evaluation, testing, work modifications, and the development of appropriate workplace policies.
Taylor TK et al., 2021 [61]	USA	ACOEM	N/A	Guideline	To provide RTW guidance for both employers and the OPS who will be supporting businesses in implementing safety measures —Part II: Industry-Specific Guidance.	Special considerations for the food industry, general office settings/warehouses, retail, healthcare, long-term care facilities, transportation and travel, construction, marine and offshore industries
Tkatek S et al., 2020 [62]	Morocco	Morocco’s Research Team System Expert	N/A	Modelling study	To develop an expert system that combines several solutions to combat COVID-19	The authors developed a methodology based on a new expert system allowing them to explore, monitor, forecast, and optimise the data collected to assist in stopping the spread of COVID-19 and make an efficient decision regarding RTW.
Zhang Q et al., 2021 [63]	China	A Chinese enterprise	500 Employees	Case Model Study	To elaborate pandemic prevention measures in a Chinese company during the RTW stage.	Description of RTW measures (i.e., Employee Information Report, Flexible Work Resumption, Health Education); the authors underlined three preconditions that could influence the RTW phase (social culture; national/local OSH-regulation and temporary guidelines for pandemic prevention; OSH practice, resources, and physical environment at the company level)
Zhao et al., 2020 [64]	China	National Health Commission of the People’s Republic of China; the Shanghai Municipal Health Commission	Not specified	Modelling study	To estimate the COVD-19 transmission dynamics under various COVID-19 prevention and control policies and offer evidence-based outcomes for RTW policies.	The authors highlighted 4 RTW policy approaches to prevent a secondary COVID-19 outbreak. The combination of quarantined and staged approaches is the most conservative and safest policy from a disease control perspective. The dynamic systems model designed in the study can serve as a tool to test various RTW policies, facilitating decision-making in responses to combating the COVID-19 pandemic.

**Table 2:** Selected papers for ‘Clinical Evaluation of Workers’. The table reported the characteristics and main findings of 20 articles, almost all cross-sectional, focused on the role of clinical evaluation of infected/non-infected workers in the RTW stage. The studies mainly came from China (8) and the USA (7) in different occupational settings, mainly healthcare and industrial.

Authors, Year	Country	Setting	Sample Size	Study Design	Aim of the Study	Main Findings
Du Y et al., 2020 [65]	China	Manufacturing and Service Industries	402 Workers	Cross-sectional	To explore the impact of the psychological contract on employees’ safety behaviour and provide preventive suggestions for combating the global spread of COVID-19.	The psychological contract and perceived insider status positively promote employees’ safety behaviour, while job burnout negatively affects it. The results show that employees’ conscious participation in safety behaviour plays an irreplaceable role in preventing COVID-19 and the safety of work resumption.
Frontera et al., 2021 [66]	USA	4 New York City hospitals	606 patients	Cohort study	Primary aim: To compare global functional outcomes between COVID-19 hospital survivors with and without neurological complications. Secondary purpose: To assess activities of daily living, Return to Work (RTW), cognitive function, anxiety, depression, fatigue and sleep abnormalities in COVID-19 hospital survivors with and without neurological complications	Patients with neurological complications were less likely to RTW than controls. Long-term functional outcomes would be worse among patients with neurological complications compared to age, gender and severity of illness-matched COVID-19 rules without neurological complications.
Ganz-Lord FA et al., 2020 [67]	USA	Montefiore Medical Center (New York City)	1698 Health Workers (HWs)	Cohort study	To evaluate symptoms, workforce implications, and testing patterns related to the COVID-19 pandemic among HWs.	From symptom onset until RTW, the median time for HWs who did not require hospitalisation was 15 days. Shortness of breath, fever, sore throat, and diarrhoea were significantly associated with longer durations from symptom onset to RTW. Among symptomatic HWs who had Real-time Polymerase Chain Reaction (Rt-PCR) testing during the study period, 51.9% tested positive.
Griffiths D et al., 2021 [68]	Australia	Australian Workers	1169 Workers	Cross-sectional	To determine the nature and prevalence of workers’ concerns regarding workplace reopening and to identify characteristics of workers and industries where particular concerns are more common	82.4% of workers reported concerns about workplace infection risk (common for HWs, retail, and accommodation/food service industries), and 53.4% reported concerns about work and home life (common for female workers, partners/spouses with dependent children). The prevalence of concerns is related to work and responsibilities at home. Actions that reduce the risk of workplace transmission, coupled with effective communication of infection controls, may alleviate worker concerns whilst recognising workers’ family and social circumstances.
Ladak et al., 2021 [69]	Canada	Canadian Rheumatology, Gastroenterology and Dermatology Associations	151 Physicians	Cross-sectional	To determine how physicians who frequently prescribe immunosuppressive medications are counselling patients on RTW before widespread vaccine distribution	94% were asked for RTW advice, and 33% felt informed enough to provide counselling. When patients requested a medical note, physicians provided one 25% of the time; among the most associated with notes were patient comorbidities, high-risk work, and vulnerable co-inhabitants. Conventional synthetic and biologic immunosuppressants did not prompt most physicians to provide a message. Respondents considered patient perspectives and workplace factors.
Lai R et al., 2020 [70]	China	Wuhan’s Hospital	861 HWs	Cross-sectional	To explore the level and influencing factors of help-seeking behaviour of returning to work in HWs.	More help-seeking was reported in HWs who had encountered problems after return, worked in a hospital before RTW, received Computerised Tomography scans and blood routine examination, had relatives or friends diagnosed or suspected as COVID-19, not a doctor, higher education and title, elder age, and single status.
Liu X et al., 2021 [71]	China	Hunan Cancer Hospital in Changsha	121 employed male cancer survivors	Cross-sectional	To explore whether fear of COVID-19 and fear of cancer recurrence are related to the likelihood of remaining at work following treatment in male cancer survivors.	Fear of COVID-19 and fear of cancer recurrence were negatively correlated with work sustainability. Significant interaction effects were observed between fear of COVID-19 and fear of cancer recurrence. Advanced disease stage, radiation therapy, and recently completed cancer treatment were all factors related to lower work sustainability scores.
Liu Z et al., 2020 [72]	USA	Industry Associations in the Energy Sector	333 Workers	Cross-sectional	To inform employers’ and policy makers’ decision making around the RTW during COVID-19.	Women, non-Caucasians, and employees living in multi-generational households were less willing to RTW. Childcare concerns were negatively related to willingness to return, whereas organisational strategies for mitigating COVID-19 transmission at work were positively associated with willingness to return.
Ly DP, 2021 [73]	USA	American Community Survey 2014–2018 5-year file	189,521 nurses; 51,834 physicians	Cross-sectional	To analyse age, disability, and household composition of nurses and physicians not in the workforce, highlighting the higher risk of COVID-19 related morbidity and mortality if exposed.	Over ¾ of nurses and physicians not in the labour force are aged 55, and about 15% have a disability. For female nurses and physicians not in the labour force, over half of those ages 20–54 had a child under 15 at home, and over half of those ages, 65+ had another adult 65 and over at home. These characteristics may present challenges and risks to returning.
Mehrsafar et al., 2021 [74]	Iran	Iranian Football League	90 Professional Football Players	Cross-sectional	To examine the relationship between competitive anxiety, fear/anxiety of COVID-19, and autonomic and endocrine stress responses in professional football players after returning to competition during the COVID-19 pandemic.	Somatic–cognitive anxiety is correlated with fear/anxiety of COVID-19 and the competition responses of salivary alpha-amylase and salivary cortisol.
Mohammadi F et al., 2021 [75]	Iran	Urmia Hospitals	14 Nurses	Cross-sectional	To determine the workplace challenges faced by nurses who had recovered from COVID-19.	The authors overviewed the challenges faced by the nurses after their RTW, such as declined ethical values, fear of re-infection, forgotten patients, gradually leaving the job, and corona-phobia.
Rex DK et al., 2020 [76]	USA	Endoscopy staff	106 HWs	Cross-sectional	To investigate the concerns of endoscopy staff regarding their risk of acquiring COVID-19 by returning to work.	Assuming no change in infection control measures, 66% were very or somewhat concerned about RTW. Four respondents preferred daily COVID-19 testing, 49 preferred weekly tests, and 47 said it did not matter. Assuming pre-COVID-19 infection control measures, endoscopists were more often unwilling to RTW compared to nonphysician staff (80% vs. 30%). After instituting new protective measures viewed as critical, 35% remained very or somewhat concerned. Wearing masks has resulted in the best preventive practice for 100% of endoscopists.
Rumrill P et al., 2021 [77]	USA	U.S. Dept. of Labor Office of Disability Employment Policy	4 Disable Workers	Cross-sectional	To demonstrate the use of a psychometrically sound assessment instrument and resource-driven planning procedure to help workers with disabilities resume or retain their employment.	Regarding RTW, for employees with neurological disabilities in the post-COVID-19 era, one potentially positive outcome of the pandemic may be that home-working will be more readily available, not only as a reasonable accommodation but also as an alternative for more significant numbers of employees than ever before.
Tan W et al., 2020 [78]	China	Chongqing enterprises	1323 Workers	Cross-sectional	To quantify the immediate psychological effects and identify preventive measures that determine the mental health of the workforce members returning to work.	RTW had not caused a high level of psychiatric symptoms, probably due to confidence instilled by psychoneuroimmunity prevention measures before the resumption of work. Low prevalence of anxiety, depression, stress and insomnia is reported, while 10.8% of workers received a Post-Traumatic Stress Disorder (PTSD) diagnosis after RTW. The severity of psychiatric symptoms was associated with marital status, presence of a physical sign, poor physical health and viewing RTW as a health hazard. There were no significant differences in the severity of psychiatric symptoms between workers/technicians and executives/managers; >95% reported psychoneuroimmunity prevention measures and were associated with less severe psychiatric symptoms.
Vanichkachorn G et al., 2021 [79]	USA	COVID-19 Activity Rehabilitation Program at Mayo Clinic	100 Patients	Cohort study	To describe characteristics of a series of patients reporting prolonged symptoms after infection with coronavirus.	Only 1 in 3 patients had returned to unrestricted work duty at the time of analysis. More than one-third of patients (34%) reported difficulties performing basic activities of daily living. Most of them required physical therapy, occupational therapy, or brain rehabilitation.
Wang S et al., 2021 [80]	China	Multicenter, Nationwide	42,000 Workers	Cross-sectional	To evaluate the prevalence of and risk factors associated with anxiety, depression, and insomnia symptoms during the RTW period in China.	Generally, 18.3, 14.9, and 17.9% of the participants had anxiety, depression, and insomnia symptoms, respectively, and 2.2–2.7% had severe symptoms. Engaging in outside activity once in ≥ 30 days and age 50–64 years were common risk factors for anxiety, depression and insomnia symptoms. Living in Hubei Province was a common risk factor for anxiety and insomnia symptoms. Working as frontline medical staff was another risk factor for anxiety symptoms.
Yang Q et al., 2020 [81]	China	Industrial Enterprises	526 Workers	Cross-sectional	To explore the impact of psychosocial stress caused by the COVID-19 pandemic on the work stress of returned workers and the boundary conditions for reducing work stress from the perspectives of perceived organisational support, perceived social support and pandemic awareness.	Psychosocial stress had a significant positive effect on employees’ work stress, whether in severe pandemic areas or non-severe pandemic areas; perceived organisational support can alleviate the impact of psychosocial stress on work stress. The moderating effect of pandemic awareness was only established in non-severe pandemic areas.
Yuan Z et al., 2021 [82]	China	Wuhan’s workers	485 participants	Cross-sectional	To investigate job reattachment as an antecedent of job engagement	Job reattachment in preparation for returning to work was related to greater levels of job engagement, which was associated with lower levels of work withdrawal and higher levels of personal protective equipment use and task performance.
Zanghì A et al., 2020 [83]	Italy	Tertiary Multiple Sclerosis Center in Catania, Italy	672 Patients	Cross-sectional	To assess the mental health status and RTW of multiple sclerosis patients.	RTW was associated with the presence of psychiatric concerns higher in patients who have started/switched disease-modifying treatment in the last 12 months or those with higher levels of disability. A total of 31.8% of patients resulted in clinically significant PTSD-like symptoms. Moderate-to-severe anxiety was reported by 48.6% of patients, while moderate-to-severe depression and moderate-to-severe stress were, respectively, reported by 22% and 50.9% of patients.
Zheng N et al., 2021 [84]	China	Healthcare setting in Hubei Province	83 Nurses	Cross-sectional	To understand the adaptation status of nurses after recovering from COVID-19 during RTW.	The working adaptation status of infected nurses resulted in a medium level; they had difficulties adapting to the fast pace of work after RTW and decreased concentration on their work. Age, marital status, hospital grade and type, work department, job title, and educational background had no significant effects on nurses’ job adaptability after RTW.

**Table 3:** Selected papers for ‘Testing Strategies related to RTW’. The table reported the characteristics and main findings of 12 articles (7 cohort and 5 cross-sectional studies) aimed to evaluate the most helpful testing strategies (test type, timing, and target definition) to guide the resumption to work of SARS-CoV-2 infected. All studies were carried out in a healthcare setting and came from Italy (4), the USA (3), Spain (2), and others.

Authors, Year	Country	Setting	Sample Size	Study Design	Aim of the Study	Main Findings
Cariani L et al., 2020 [85]	Italy	Ca’ Granda Ospedale Maggiore Policlinico in Milan, Italy	182 health workers (HWs)	Cohort study	To evaluate the time length of negativisation from HWs’ symptoms onset significant variations in cycle threshold (Ct) values and gene positivity among positive individuals who returned to work.	The median time length of negativisation was four weeks (35% symptomatic Vs 40% asymptomatic). Three-gene positivity had the most significant variability and increasing Ct values from single- to three-gene positivity among all age groups was observed. Self-isolation of longer than two weeks and prolonged follow-up periods could be the most suitable to reduce the SARS-CoV-2 spread.
Domeracki S et al., 2020 [86]	USA	San Francisco Veterans Affairs Health Care System	12 HWs	Cross-sectional	To ascertain whether real-time polymerase chain reaction (Rt-PCR) cycle amplifications until detection, the cycle threshold (Ct), could help inform RTW strategies for HWs recovering from COVID-19 infection.	Time elapsed until Rt-PCR test-based RTW clearance ranged from 7 to 57 days (median, 34.5 days). Lower initial Ct correlated with the total time elapsed until clearance. Thus, considering the Rt-PCR Ct, which correlates with the estimated viral load, may help inform return to work (RTW) planning and decision making.
Garzaro G. et al., 2020 [87]	Italy	Città della Salute e della Scienza di Torino University- Hospital in Turin, Italy	2,411 HWs	Cross sectional	To evaluate the early impact of structured risk management for exposed COVID-19 HWs and describe how their characteristics contributed to infection and diffusion.	Among 830 HWs who were at ‘high/medium risk’, 9.6% tested positive. Physicians and non-medical services resulted in an increased risk. Patient care did not increase the risk but sharing the work environment did. HWs with management positions were the main source of infection due to the high number of interactions.
Gombar S at al., 2020 [88]	USA	Stanford Healthcare	63 HWs / 87 patients	Cohort study	To understand the appropriate length of symptom to determine RTW and contact precaution strategies.	The average time to transition from Rt-PCR positive to negative was 24 days after symptom onset. A total of 20% of individuals remain Rt-PCR positive for more than one month from symptom onset, and 10% of the patients did not have a negative test until after 33 days had passed. These findings suggest that the fixed length of time before returning to work be revised to over one month.
González Martin -Moro G et al., 2021 [89]	Spain	Henares University Hospital in Coslada, Madrid, Spain	374 HWs	Cohort study	To determine the most efficient time to perform Rt-PCR prior HWs resumption.	The median time to negativisation was 25 days from symptom onset (IQR 20–35 days). Some clinical variables (dyspnoea, cough) were correlated with longer times to negativisation and may be considered in developing RTW protocols. Rt-PCR during the first three weeks leads to a high percentage of positive results. In the presence of respiratory symptoms, negativisation took nearly one week more.
Guarnieri V et al., 2021 [90]	Italy	Meyer Children’s University Hospital in Florence, Italy	1690 HWs: Screening 1472 Contacts 188 RTW 30	Cross-sectional	To describe a healthcare surveillance experience based on a combined screening consisting of Rt-PCR on nasopharyngeal swabs and rapid serologic tests for SARS-CoV-2.	A total of 13/1690 without clinical manifestations was found positive for SARS-CoV-2 using Rt-PCR: 8/1472 were found positive during the screening, 1/188 during contact with a positive individual, while 4/30 were found positive on the day of re-admission at work after an influenza-like illness. Concerning working areas, most Rt-PCR positivity and se*rologic positivity were found in non-COVID-19 dedicated areas. No cases were registered among non-patient-facing workers. Nurses and residents represented, respectively, the working roles with the highest and lowest percentage of Rt-PCR positivity.
Pan SC et al., 2021 [91]	Taiwan	National Taiwan University Hospital in Taipei, Taiwan	14,210 HWs	Cohort study	To describe experience implementing specific infection prevention and control policy and practice during the first six months of the pandemic.	Among 14,210 HWs, there were 367 (2.6%) incident events (with one or more predefined symptoms during a reporting interval). A total of 283 HWs were tested for COVID-19; 179 had predefined symptoms, and 104 were asymptomatic. Many of the tests (59.7%) were performed as part of the Extended COVID-19 Screening Program. Hospital-wide web-based health surveillance integrated with a risk-based management algorithm and molecular testing of asymptomatic HWs allowed authors to rapidly identify workers at risk of infection and prevent spread to other HWs and patients.
Porru S et al., 2020 [92]	Italy	University Hospital of Verona, Italy	5942 HWs	Cross-sectional	To report a SARS-CoV-2mass test experience among HWs population, as part of risk assessment and management pandemic program.	Positive tests were returned for 238 workers, similarly in COVID and non-COVID units. The SARS-CoV-2 risk was not affected by gender, age, or job type, whereas work setting and occupation were both predictors of infection. The risk was higher in medical wards and health services and lower in surgical wards and administration areas. Mass screening improved risk assessment, limited SARS-CoV-2 diffusion, and allowed resumption to work for infected HW.
Rivett L et al., 2020 [93]	U.K.	Cambridge University Hospitals NHS Foundation Trust, UK	1032 HWs	Cross-sectional	To highlight challenges to the roll-out of expanded screening programs.	A total of 1032 asymptomatic HWs were screened for SARS-CoV-2 over three weeks. Symptomatic staff and symptomatic household contacts were additionally tested. Thirty HWs in the asymptomatic screening group tested positive; 57% were truly asymptomatic/paucisymptomatic, while about 40% had experienced symptoms >7 days before testing. Clusters of HWs infection were discovered on two independent wards. These data supported the utility of comprehensive screening of HWs, with minimal or no symptoms, for protecting patients and hospital staff.
Shenoy ES et al., 2020 [94]	USA	Massachusetts General Brigham (MGB), USA	8930 Employees	Cohort study	To evaluate average intervals until test-based clearance and the number of excesses lost workdays using test-based authorisation.	One thousand and forty-nine employees were positive for SARS-CoV-2; 37 (3.5%) were hospitalised within seven days of their positive test. The median number of days from the first positive to the first negative was 17 (range 2-38 days). Of the 425 HWs with positive SARS-CoV-2 test results, 263 (61.9%) had a sequential second negative NP. The median number of days from the first positive to the second negative was 19 (range 6–37). If test-based criteria are used for RTW, the authors recommend establishing a minimum duration of days before the clearance test. Test-based clearance accounted for an additional 4,097 days of cumulative lost work time, corresponding to a mean of 7.2 extra days of work lost per employee than would have been accrued using the time plus symptom-based clearance method. Thus, switching to time plus symptom-based clearance criteria could allow an earlier RTW for most workers and aid in workforce preservation.
Tripathy D et al., 2021 [95]	India	Tertiary eye care facility in Odisha, India	87 HWs /224 patients	Cohort study	To report the use and impact of a point-of-care Rapid Antigen Test in facilitating commencement of elective surgeries and contact tracing of exposed HWs and implement RTW policy.	The overall positivity rate was around 7%. Asymptomatic patients screened preoperatively had a lower positivity rate at about 3% than the staff (who were either known contacts or were symptomatic) at around 17%. Contact tracing found three-quarters of the staff at low risk and only one quarter at medium or high risk. Rapid Antigenic Tests may be routinely considered for indication-based preoperative screening of asymptomatic patients and on-campus screening, contact tracing and implementation of RTW policies for HWs.
Villarreal J et al., 2021 [96]	Spain	Fundacion Jimenez Dıaz University Hospital in Madrid, Spain	375 HWs	Cohort study	To investigate whether HWs’ RTW after COVID-19 was associated with time to a negative viral detection test.	A delayed RTW was associated with longer intervals (>30 days) to a negative Rt-PCR after symptom onset and age, sex, and nursing staff and clinical support services compared to physicians. A predictive model based on those variables is proposed.

## Data Availability

The data presented in this study are available on request from the corresponding author.

## References

[1] World Health Organization (WHO). https://covid19.who.int.

[2] Wei Y., Guan J., Ning X., Li Y., Wei L., Shen S., Zhang R., Zhao Y., Shen H., Chen F. (2021). Global COVID-19 pandemic waves: Limited lessons learned worldwide over the past year. Engineering.

[3] Alwan N.A., Burgess R.A., Ashworth S., Beale R., Bhadelia N., Bogaert D., Dowd J., Eckerle I., Goldman L.R., Greenhalgh T. (2020). Scientific consensus on the COVID-19 pandemic: We need to act now. Lancet.

[4] Marziano V., Guzzetta G., Rondinone B.M., Boccuni F., Riccardo F., Bella A., Poletti P., Trentini F., Pezzotti P., Brusaferro S. (2021). Retrospective analysis of the Italian exit strategy from COVID-19 lockdown. Proc. Natl. Acad. Sci. USA.

[5] Sugerman-Brozan J. (2020). Health Technical Committee of the Massachusetts Coalition for Occupational Safety and Health. Measures to Protect the Health and Safety of Massachusetts Employees Who Must Work at the Workplace During the SARS-CoV-2 Pandemic. New Solut..

[6] Marcone V. (2020). Reduction of Contagion Risks by SARS-Cov-2 (COVID-19) in Air-Conditioned Work Environments. Pain Physician.

[7] Garzillo E.M., Monaco M.G.L., Spacone A., Inglese E., Lamberti M., Pompei D. (2020). SARS-CoV-2 emergency in the workplace: Are companies ready to protect their workers? A cross-sectional survey. Int. J. Occup. Saf. Ergon..

[8] Rueda-Garrido J.C., Vicente-Herrero M.T., Del Campo M.T., Reinoso-Barbero L., de la Hoz R.E., Delclos G.L., Kales S.N., Fernandez-Montero A. (2020). Return to work guidelines for the COVID-19 pandemic. Occup. Med..

[9] Shi L., Lu Z.-A., Que J.-Y., Huang X.-L., Lu Q.-D., Liu L., Zheng Y.-B., Liu W.-J., Ran M.-S., Yuan K. (2021). Long-Term Impact of COVID-19 on Mental Health among the General Public: A Nationwide Longitudinal Study in China. Int. J. Environ. Res. Public Health.

[10] Torres A.E., Ozog D.M., Hruza G.J. (2021). Coronavirus Disease 2019 and Dermatology Practice Changes. Dermatol. Clin..

[11] Rizk H.G., Strange C., Atallah S., Massingale S., Clendaniel R. (2020). Coronavirus Disease 2019 Return to Work Guidance and Recommendations for Vestibular Clinicians. Ear Hear..

[12] Lakkireddy D.R., Chung M.K., Deering T.F., Gopinathannair R., Albert C.M., Epstein L.M., Harding C.V., Hurwitz J.L., Jeffery C.C., Krahn A.D. (2020). Guidance for Rebooting Electrophysiology Through the COVID-19 Pandemic From the Heart Rhythm Society and the American Heart Association Electrocardiography and Arrhythmias Committee of the Council on Clinical Cardiology: Endorsed by the American College of Cardiology. JACC Clin. Electrophysiol..

[13] Benito D.A., Pasick L.J., Mulcahy C.F., Rajasekaran K., Todd-Hesham H., Joshi A.S., Goodman J.F., Thakkar P. (2020). Local spikes in COVID-19 cases: Recommendations for maintaining otolaryngology clinic operations. Am. J. Otolaryngol..

[14] Geneid A., Nawka T., Schindler A., Oguz H., Chrobok V., Calcinoni O., Am Zehnhoff-Dinnesen A., Neumann K., Farahat M., Abou-Elsaad T. (2020). Union of the European Phoniatricians’ position statement on the exit strategy of phoniatric and laryngological services: Staying safe and getting back to normal after the peak of coronavirus disease 2019 (issued on 25th May 2020). J. Laryngol. Otol..

[15] Soffin E.M., Reisener M.J., Sama A.A., Beckman J.D., Liguori G.A., Lebl D.R., Girardi F.P., Cammisa F.P., Hughes A.P. (2020). Essential Spine Surgery During the COVID-19 Pandemic: A Comprehensive Framework for Clinical Practice from a Specialty Orthopedic Hospital in New York City. HSS J..

[16] Nobel T.B., Marin M., Divino C.M. (2020). Lessons in flexibility from a general surgery program at the epicenter of the pandemic in New York City. Surgery.

[17] Juprasert J.M., Gray K.D., Moore M.D., Obeid L., Peters A.W., Fehling D., Fahey T.J., Yeo H.L. (2020). Restructuring of a General Surgery Residency Program in an Epicenter of the Coronavirus Disease 2019 Pandemic: Lessons From New York City. JAMA Surg..

[18] de Amorim L.M., Maske T.T., Ferreira S.H., Dos Santos R.B., Feldens C.A., Kramer P.F. (2020). New Post-COVID-19 Biosafety Protocols in Pediatric Dentistry. Pesqui Bras. Odontopediatria Clín. Integr..

[19] Moura-Neto J.A., Palma L.M.P., Marchiori G.F., Stucchi R.S.B., Misael A.M., D’Avila R., Silva D.R.D., Andreoli M.C.C., Kraychete A., Bastos K. (2020). Recommendations from the Brazilian Society of Nephrology for approaching Covid-19 Diagnostic Testing in Dialysis Units. J. Bras. Nefrol..

[20] Jin S., He Y., Yang K., Gan Q., Huang W., Wang X., Meng C., Wang H. (2021). The Resumption of Sports Medicine During the COVID-19 Post-Epidemic Period: Experiences from Wuhan, People’s Republic of China. J. Bone Joint Surg. Am..

[21] Wilson K.C., Kaminsky D.A., Michaud G., Sharma S., Nici L., Folz R.J., Barjaktarevic I., Bhakta N.R., Cheng G., Chupp G.L. (2020). Restoring Pulmonary and Sleep Services as the COVID-19 Pandemic Lessens. From an Association of Pulmonary, Critical Care, and Sleep Division Directors and American Thoracic Society-coordinated Task Force. Ann. Am. Thorac. Soc..

[22] Sunandar H., Ramdhan D.K. (2021). Preventing and Controlling COVID-19: A Practical-Based Review in Offshore Workplace. Kesmas Jurnal Kesehatan Masyarakat Nasional.

[23] Callander D., Meunier É., DeVeau R., Grov C., Donovan B., Minichiello V., Singham Goodwin A., Duncan D.T. (2020). Sex workers are returning to work and require enhanced support in the face of COVID-19: Results from a longitudinal analysis of online sex work activity and a content analysis of safer sex work guidelines. Sex Health.

[24] Simić N., Stefanović M., Petrović G., Stanković A. (2021). Use of the risk analysis approach n the Serbian army integration process against Covid-19. Oper. Res. Eng. Sci. Theor. Appl..

[25] Falorca J.F. (2021). Envisioning a strategic framework to streamline building operation, sustainability and users’ disease control. J. Facil. Manag..

[26] Iavicoli S., Boccuni F., Buresti G., Gagliardi D., Persechino B., Valenti A., Rondinone B.M. (2021). Risk assessment at work and prevention strategies on COVID-19 in Italy. PLoS ONE.

[27] Carvalhais C., Querido M., Pereira C.C., Santos J. (2021). Biological risk assessment: A challenge for occupational safety and health practitioners during the COVID-19 (SARS-CoV-2) pandemic. Work.

[28] Binnicker M.J. (2020). Can the Severe Acute Respirat.tory Syndrome Coronavirus 2 Polymerase Chain Reaction Cycle Threshold Value and Time From Symptom Onset to Testing Predict Infectivity?. Clin. Infect. Dis..

[29] Longtin Y., Charest H., Quach C., Savard P., Baz M., Boivin G., Farfard J., Villeneuve J., Roger M., De Serres G. (2021). Infectivity of healthcare workers diagnosed with coronavirus disease 2019 (COVID-19) approximately 2 weeks after onset of symptoms: A cross-sectional study. Infect. Control. Hosp. Epidemiol..

[30] Stewart-Patterson C., Bourgeois R., Martin D.W. (2021). The Importance of Keeping Patients with Post-Acute Sequelae of SARS-CoV-2 Infection (Long COVID) Engaged in Work. Am. Fam. Physician.

[31] Cavasin D., Paladino M.E., Riva M.A., Persico G., Belingheri M. (2021). Prolonged PCR Positivity Stigma and Return-To-Work After SARS-CoV-2 Infection. J. Occup. Environ. Med..

[32] Chua K.Y., Holmes N.E., Kwong J. (2020). Prolonged PCR positivity in health care workers with COVID-19: Implications for practice guidelines. Med. J. Aust.

[33] Henderson D.K., Weber D.J., Babcock H., Hayden M.K., Malani A., Wright S.B., Murthy A.R., Guzman-Cottrill J., Haessler S., Rock C. (2021). The perplexing problem of persistently PCR-positive personnel. Infect. Control. Hosp. Epidemiol..

[34] Caban-Martinez A.J., Schaefer-Solle N., Santiago K., Louzado-Feliciano P., Brotons A., Gonzalez M., Issenberg S.B., Kobetz E. (2020). Epidemiology of SARS-CoV-2 antibodies among firefighters/paramedics of a US fire department: A cross-sectional study. Occup. Environ. Med..

[35] Krsak M., Johnson S.C., Poeschla E.M. (2020). COVID-19 Serosurveillance May Facilitate Return-to-Work Decisions. Am. J. Trop. Med. Hyg..

[36] Ray S., Chawla N., Gupta A., Maramaraj K.K., Kumar S., Anand K.B. (2020). Return to work strategy with antibody-based tests in COVID19: An observational study from a metropolitan area, India. J. Mar. Med. Soc..

[37] Saretto G., Bozzi C. (2021). A proposal for the management of health surveillance and monitoring procedures in relation to the risks posed by SARS-COV-2 to hospital and nursing home workers, based on strategies implemented in the facilities of the Fondazione Opera San Camillo. G Ital. Med. Lav. Ergon..

[38] Leso V., Fontana L., Iavicoli I. (2021). Susceptibility to Coronavirus (COVID-19) in Occupational Settings: The Complex Interplay between Individual and Workplace Factors. Int. J. Environ. Res. Public Health.

[39] Huyck K.L., McDonough C.M., Kennedy D.D., Phillips P., Haig A.J. (2021). Return to Work in the Pandemic—Considerations beyond Infection. PM R.

[40] Moher D., Liberati A., Tetzlaff J., Altman D.G. (2009). PRISMA Group. Preferred reporting items for systematic reviews and meta-analyses: The PRISMA statement. J. Clin. Epidemiol..

[41] Schardt C., Adams M.B., Owens T., Keitz S., Fontelo P. (2007). Utilization of the PICO framework to improve searching PubMed for clinical questions. BMC Med. Inform. Decis. Mak..

[42] https://www.zotero.org/.

[43] https://www.rayyan.ai/.

[44] Microsoft Corporation (2018). Microsoft Excel [Internet]. https://office.microsoft.com/excel.

[45] VOSVIEWER. https://www.vosviewer.com.

[46] Barriga Medina H.R., Campoverde Aguirre R., Coello-Montecel D., Ochoa Pacheco P., Paredes-Aguirre M.I. (2021). The Influence of Work-Family Conflict on Burnout during the COVID-19 Pandemic: The Effect of Teleworking Overload. Int. J. Environ. Res. Public Health.

[47] Brosseau L.M., Rosen J., Harrison R. (2021). Selecting Controls for Minimizing SARS-CoV-2 Aerosol Transmission in Workplaces and Conserving Respiratory Protective Equipment Supplies. Ann. Work Expo Health.

[48] Calderwood M.S., Deloney V.M., Anderson D.J., Cheng V.C., Gohil S., Kwon J.H., Mody L., Monsees E., Vaughn V.M., Wiemken T.L. (2020). Policies and practices of SHEA Research Network hospitals during the COVID-19 pandemic. Infect. Control. Hosp. Epidemiol..

[49] Expósito-Delgado A.J., Ausina-Márquez V., Mateos-Moreno M.V., Martínez-Sanz E., Del Carmen Trullols-Casas M., Llamas-Ortuño M.E., Blanco-González J.M., Almerich-Torres T., Bravo M., Martínez-Beneyto Y. (2021). Delivery of Health Care by Spanish Dental Hygienists in Private and Public Dental Services during the COVID-19 De-Escalation Phase (June 2020): A Cross-Sectional Study. Int. J. Environ. Res. Public Health.

[50] Ge Y., Zhang W.B., Wang J., Liu M., Ren Z., Zhang X., Zhou C., Tian Z. (2021). Effect of different resumption strategies to flatten the potential COVID-19 outbreaks amid society reopens: A modeling study in China. BMC Public Health.

[51] Gross D.P., Asante A., Pawluk J., Niemeläinen R. (2021). A Descriptive Study of the Implementation of Remote Occupational Rehabilitation Services Due to the COVID-19 Pandemic within a Workers’ Compensation Context. J. Occup. Rehabil..

[52] Lichtman A., Greenblatt E., Malenfant J., Kuo A. (2021). Universal symptom monitoring to address presenteeism in healthcare workers. Am. J. Infect. Control.

[53] Lu Y., Wu J., Peng J., Lu L. (2020). The perceived impact of the Covid-19 epidemic: Evidence from a sample of 4807 SMEs in Sichuan Province, China. Environ. Hazards.

[54] Marzban S., Durakovic I., Candido C., Mackey M. (2021). Learning to work from home: Experience of Australian workers and organizational representatives during the first Covid-19 lockdowns. J. Corp Real Estate.

[55] Onesti C.E., Rugo H.S., Generali D., Peeters M., Zaman K., Wildiers H., Harbeck N., Martin M., Cristofanilli M., Cortes J. (2020). Oncological care organisation during COVID-19 outbreak. ESMO Open.

[56] Pratama M.R., Supriyadi A., Sari N. (2021). Assessment of Precautionary Measures against COVID-19 in Indonesian Workplaces. Int. J. Public Health Sci..

[57] Robinson J., Kengatharan N. (2020). Exploring the effect of Covid-19 on Small and Medium Enterprises: Early Evidence from Sri Lanka. J. Appl. Econ. Bus. Res..

[58] Salgarello S., Salvadori M., Mazzoleni F., Francinelli J., Bertoletti P., Audino E., Garo M.L. (2021). The New Normalcy in Dentistry after the COVID-19 Pandemic: An Italian Cross-Sectional Survey. Dent. J..

[59] Soneru C.N., Fernandez A.M., Bradford V., Staffa S.J., Raman V.T., Cravero J., Zurakowski D., Meier P.M. (2021). Pediatric Anesthesia COVID-19 Collaborative. A survey of the global impact of COVID-19 on the practice of pediatric anesthesia: A study from the pediatric anesthesia COVID-19 Collaborative Group. Paediatr Anaesth..

[60] Taylor T.K., Das R., Mueller K., Pransky G., Christian J., Orford R., Blink R. (2020). Safely Returning America to Work: Part I: General Guidance for Employers. J. Occup. Environ. Med..

[61] Taylor T.K., Das R., Mueller K.L., Pransky G.S., Harber P., McLellan R.K., Hartenbaum N.P., Behrman A.J., Roy D.R., Blink R.C. (2021). Safely Returning America to Work Part II: Industry-Specific Guidance. J. Occup. Environ. Med..

[62] Tkatek S., Belmzoukia A., Nafai S., Abouchabaka J., Ibnou-Ratib Y. (2020). Putting the world back to work: An expert system using big data and artificial intelligence in combating the spread of COVID-19 and similar contagious diseases. Work.

[63] Zhang Q., Wu Y., Li M., Li L. (2021). Epidemic Prevention During Work Resumption: A Case Study of One Chinese Company’s Experience. Front. Public Health.

[64] Zhao J., Jia J., Qian Y., Zhong L., Wang J., Cai Y. (2020). COVID-19 in Shanghai: IPC Policy Exploration in Support of Work Resumption Through System Dynamics Modeling. Risk Manag. Healthc. Policy.

[65] Du Y., Liu H. (2020). Analysis of the Influence of Psychological Contract on Employee Safety Behaviors against COVID-19. Int. J. Environ. Res. Public Health.

[66] Frontera J.A., Yang D., Lewis A., Patel P., Medicherla C., Arena V., Fang T., Andino A., Snyder T., Madhavan M. (2021). A prospective study of long-term outcomes among hospitalized COVID-19 patients with and without neurological complications. J. Neurol. Sci..

[67] Ganz-Lord F.A., Segal K.R., Rinke M.L. (2021). COVID-19 symptoms, duration, and prevalence among healthcare workers in the New York metropolitan area. Infect. Control. Hosp. Epidemiol..

[68] Griffiths D., Sheehan L., van Vreden C., Whiteford P., Collie A. (2021). Returning to the Workplace during the COVID-19 Pandemic: The Concerns of Australian Workers. J. Occup. Rehabil..

[69] Ladak K., Winthrop K., Marshall J.K., Gelfand J., Pope J. (2021). Counselling patients for return to work on immunosuppression: Practices of Canadian specialists during the COVID-19 pandemic. Clin. Exp. Rheumatol..

[70] Lai R., Tan L., Lai X., Zhang X., Zhou Q. (2020). Help-Seeking Behavior of Returning to Work in Healthcare Workers and its Influencing Factors During COVID-19 Subsiding. J. Occup. Environ. Med..

[71] Liu X., Cheng A.S., Zeng Y., Zhang X., Peng X., Hu H., Li H., Feuerstein M. (2021). Fears of COVID-19 and cancer recurrence related to work sustainability among male cancer survivors. J. Mens Health.

[72] Liu Z., Van Egdom D., Flin R., Spitzmueller C., Adepoju O., Krishnamoorti R. (2020). I Don’t Want to Go Back: Examining the Return to Physical Workspaces During COVID-19. J. Occup. Environ. Med..

[73] Ly D.P. (2021). Age, disability, and household composition of nurses and physicians who are not in the labor force. PLoS ONE.

[74] Mehrsafar A.H., Moghadam Zadeh A., Jaenes Sánchez J.C., Gazerani P. (2021). Competitive anxiety or Coronavirus anxiety? The psychophysiological responses of professional football players after returning to competition during the COVID-19 pandemic. Psychoneuroendocrinology.

[75] Mohammadi F., Radfar M., Hemmati Maslak Pak M. (2021). Workplace challenges and nurses recovered from COVID-19. Nurs. Ethics.

[76] Rex D.K., Vemulapalli K.C., Lahr R.E., McHenry L., Sherman S., Al-Haddad M. (2020). Endoscopy Staff Are Concerned About Acquiring Coronavirus Disease 2019 Infection When Resuming Elective Endoscopy. Gastroenterology.

[77] Rumrill P., Rumrill S., Sheppard-Jones K., Rumrill A., Graham-Smith M., Curry B., Wiley L., Fisher E., Kabeya A., Adams C. (2021). Identifying the Job Accommodation Needs of American Workers with Mid-career Neurological Disabilities: A Multiple Case Study Investigation. J. Vocat Rehabil..

[78] Tan W., Hao F., McIntyre R.S., Jiang L., Jiang X., Zhang L., Zhao X., Zou Y., Hu Y., Luo X. (2020). Is returning to work during the COVID-19 pandemic stressful? A study on immediate mental health status and psychoneuroimmunity prevention measures of Chinese workforce. Brain Behav. Immun..

[79] Vanichkachorn G., Newcomb R., Cowl C.T., Murad M.H., Breeher L., Miller S., Trenary M., Neveau D., Higgins S. (2021). Post-COVID-19 Syndrome (Long Haul Syndrome): Description of a Multidisciplinary Clinic at Mayo Clinic and Characteristics of the Initial Patient Cohort. Mayo Clin. Proc..

[80] Wang S., Zhang Y., Guan Y., Ding W., Meng Y., Hu H., Liu Z., Zeng X., Wang M. (2021). A nationwide evaluation of the prevalence of and risk factors associated with anxiety, depression and insomnia symptoms during the return-to-work period of coronavirus disease 2019 in China. Soc. Psychiatry Psychiatr Epidemiol..

[81] Yang Q., Huo J., Li J., Jiang Y. (2020). Research on the influence of the COVID-19 epidemic on work stress of returning workers in China: A study based on empirical analyses of industrial enterprises. Work.

[82] Yuan Z., Ye Z., Zhong M. (2021). Plug back into work, safely: Job reattachment, leader safety commitment, and job engagement in the COVID-19 pandemic. J. Appl. Psychol..

[83] Zanghì A., D’Amico E., Luca M., Ciaorella M., Basile L., Patti F. (2020). Mental health status of relapsing-remitting multiple sclerosis Italian patients returning to work soon after the easing of lockdown during COVID-19 pandemic: A monocentric experience. Mult. Scler. Relat. Disord..

[84] Zheng N., Zhang T., Liu Y., Zhu X.Q. (2021). Investigation of the Status of Nurses Returning to Work After Recovering From COVID-19 and Influencing Factors. J. Nurs. Care Qual..

[85] Cariani L., Orena B.S., Ambrogi F., Gambazza S., Maraschini A., Dodaro A., Oggioni M., Orlandi A., Pirrone A., Uceda Renteria S. (2020). Time Length of Negativization and Cycle Threshold Values in 182 Healthcare Workers with Covid-19 in Milan, Italy: An Observational Cohort Study. Int. J. Environ. Res. Public Health.

[86] Domeracki S., Clapp R.N., Taylor K., Lu C.M., Lampiris H., Blanc P.D. (2020). Cycle Threshold to Test Positivity in COVID-19 for Return to Work Clearance in Health Care Workers. J. Occup. Environ. Med..

[87] Garzaro G., Clari M., Ciocan C., Grillo E., Mansour I., Godono A., Borgna L.G., Sciannameo V., Costa G., Raciti I.M. (2020). COVID-19 infection and diffusion among the healthcare workforce in a large university-hospital in northwest Italy. Med. Lav..

[88] Gombar S., Chang M., Hogan C.A., Zehnder J., Boyd S., Pinsky B.A., Shah N.H. (2020). Persistent detection of SARS-CoV-2 RNA in patients and healthcare workers with COVID-19. J. Clin. Virol..

[89] González Martin-Moro J., Chamorro Gómez M., Dávila Fernández G., Elices Apellaniz A., Fernández Hortelano A., Guzmán Almagro E., Herranz Varela A., Izquierdo Rodríguez C., Molina Montes B., Sánchez Moreno G.V. (2021). Survival analysis of time to SARS-CoV-2 PCR negativisation to optimise PCR prescription in health workers: The Henares COVID-19 healthcare workers cohort study. Occup. Environ. Med..

[90] Guarnieri V., Moriondo M., Giovannini M., Lodi L., Ricci S., Pisano L., Barbacci P., Bini C., Indolfi G., Zanobini A. (2021). Surveillance on Healthcare Workers During the First Wave of SARS-CoV-2 Pandemic in Italy: The Experience of a Tertiary Care Pediatric Hospital. Front. Public Health.

[91] Pan S.C., Hsu M.C., Chang H.H., Wang J.T., Lai Y.L., Chen P.C., Chang S.Y., Sheng W.H., Chen Y.C., Chen S.C. (2021). Prospective health surveillance for COVID-19 among health care workers at a university medical center in Taiwan, January to June 2020. J. Formos Med. Assoc..

[92] Porru S., Carta A., Monaco M.G.L., Verlato G., Battaggia A., Parpaiola M., Lo Cascio G., Pegoraro M., Militello V., Moretti F. (2020). Health Surveillance and Response to SARS-CoV-2 Mass Testing in Health Workers of a Large Italian Hospital in Verona, Veneto. Int. J. Environ. Res. Public Health.

[93] Rivett L., Sridhar S., Sparkes D., Routledge M., Jones N.K., Forrest S., Young J., Pereira-Dias J., Hamilton W.L., Ferris M. (2020). Screening of healthcare workers for SARS-CoV-2 highlights the role of asymptomatic carriage in COVID-19 transmission. Elife.

[94] Shenoy E.S., West L.R., Hooper D.C., Sheehan R.R., Hashimoto D., Boukus E.R., Aurora M.N., McEvoy D.S., Klompas M. (2020). Healthcare worker infection with SARS-CoV-2 and test-based return to work. Infect. Control. Hosp. Epidemiol..

[95] Tripathy D., Roy A.K., Khanna R.C., Jalali S., Panigrahy B., Parija D.C., Rath S. (2021). Point-of-care rapid antigen testing for COVID-19 at a tertiary eye care facility: Role in commencement of elective surgeries, contact tracing and implementation of back-to-work policy. Indian J. Ophthalmol..

[96] Villarreal J., Nieto S.V., Vázquez F., Del Campo M.T., Mahillo I., de la Hoz R.E. (2021). Time to a Negative SARS-CoV-2 PCR Predicts Delayed Return to Work After Medical Leave in COVID-19 Infected Health Care Workers. J. Occup. Environ. Med..

[97] Li Z., Li G., He J., Cao D., Tian J. (2021). The Smart Safeguard System for COVID-19 to prevent cluster-infection in workplaces. J. Infect. Public Health.

[98] Niu Q., Nagata T., Fukutani N., Tezuka M., Shimoura K., Nagai-Tanima M., Aoyama T. (2021). Health effects of immediate telework introduction during the COVID-19 era in Japan: A cross-sectional study. PLoS ONE.

[99] Wood S.J., Michaelides G., Inceoglu I., Hurren E.T., Daniels K., Niven K. (2021). Homeworking, Well-Being and the COVID-19 Pandemic: A Diary Study. Int. J. Environ. Res. Public Health.

[100] Radonić M., Vukmirović V., Milosavljević M. (2021). The Impact of Hybrid Workplace Models on Intangible Assets: The Case of an Emerging Country. Amfiteatru Econ..

[101] World Health Organization (2010). Telemedicine: Opportunities and developments in Member States: Report on the second global survey on eHealth 2009. (Global Observatory for eHealth Series, 2). https://www.who.int/goe/publications/goe_telemedicine_2010.pdf.

[102] Ashry A.H., Alsawy M.F. (2020). Doctor-patient distancing: An early experience of telemedicine for postoperative neurosurgical care in the time of COVID-19. Egypt J. Neurol. Psychiatr. Neurosurg..

[103] Salazar M.Á., Chavez-Galan L., Castorena-Maldonado A., Mateo-Alonso M., Diaz-Vazquez N.O., Vega-Martínez A.M., Martínez-Orozco J.A., Becerril-Vargas E., Sosa-Gómez F.M., Patiño-Gallegos H. (2021). Low Incidence and Mortality by SARS-CoV-2 Infection Among Healthcare Workers in a Health National Center in Mexico: Successful Establishment of an Occupational Medicine Program. Front. Public Health.

[104] Trivedy C., Mills I., Dhanoya O. (2020). The impact of the risk of COVID-19 on Black, Asian and Minority Ethnic (BAME) members of the UK dental profession. Br. Dent. J..

[105] Banaee S., Claiborne D.M., Akpinar-Elci M. (2021). Occupational health practices among dental care professionals before and during the COVID-19 pandemic. Work.

[106] Manucci M. (2021). How People Come Back to Workplaces during the Pandemic: Three dimensions of intervention for new emotional performance conditions. Hum. Resour. Dev. Int..

[107] Sarihasan I., Oláh J., Al-Dalahmeh M., Yousuf A., Dajnoki K. (2021). Determining the importance of high-reliability healthcare organizations during the Covid-19 pandemic: Evidence from healthcare workers in Turkey. Probl. Perspect. Manag..

[108] Sizemore L.M., Peganoff-O’Brien S., Skubik-Peplaski C. (2021). Interference: COVID-19 and the Impact on Potential and Performance in Healthcare. Work.

[109] Trivedi A., Fontelera M., Ishak N., Lai A., Win K.N., Ismail K., Koh D. (2021). Healthcare workers’ preparedness and response during COVID-19 pandemic. Proc. Singap. Healthc..

[110] Soriano J.B., Murthy S., Marshall J.C., Relan P., Diaz J.V. (2021). WHO Clinical Case Definition Working Group on Post-COVID-19 Condition. A clinical case definition of post-COVID-19 condition by a Delphi consensus. Lancet Infect. Dis..

[111] Asadi-Pooya A.A., Akbari A., Emami A., Lotfi M., Rostamihosseinkhani M., Nemati H., Barzegar Z., Kabiri M., Zeraatpisheh Z., Farjoud-Kouhanjani M. (2021). Long COVID syndrome-associated brain fog. J. Med. Virol..

[112] Fan C.Y., Fann J.C., Yang M.C., Lin T.Y., Chen H.H., Liu J.T., Yang K.C. (2021). Estimating global burden of COVID-19 with disability-adjusted life years and value of statistical life metrics. J. Formos. Med. Assoc..

[113] Brown N., Nicholson J., Campbell F.K., Patel M., Knight R., Moore S. (2021). COVID-19 Post-lockdown: Perspectives, implications and strategies for disabled staff. Alter-Eur. J. Disabil. Res..

[114] Jones J.M., Saeed H., Katz M.S., Lustberg M.B., Forster V.J., Nekhlyudov L. (2021). Readdressing the Needs of Cancer Survivors During COVID-19: A Path Forward. J. Natl. Cancer Inst..

[115] Scheid J.L., Lupien S.P., Ford G.S., West S.L. (2020). Commentary: Physiological and Psychological Impact of Face Mask Usage during the COVID-19 Pandemic. Int. J. Environ. Res. Public Health.

[116] Iddins B.O., Waugh M.H., Buck B., Cato T., Graham D.E., Attia K., Jones D., Partin A., Shourbaji R., Wesh C. (2021). Benchmarking SARS CoV-2 Infection in the Workplace to Support Continuity of Operations. J. Occup. Environ. Med..

[117] Oliveira B.A., Oliveira L.C., Sabino E.C., Okay T.S. (2020). SARS-CoV-2 and the COVID-19 disease: A mini review on diagnostic methods. Rev. Inst. Med. Trop. Sao Paulo.

[118] Makoah N.A., Tipih T., Litabe M.M., Brink M., Sempa J.B., Goedhals D., Burt F.J. (2021). A systematic review and meta-analysis of the sensitivity of antibody tests for the laboratory confirmation of COVID-19. Future Virol..

[119] Rostami A., Sepidarkish M., Leeflang M.M.G., Riahi S.M., Nourollahpour Shiadeh M., Esfandyari S., Mokdad A.H., Hotez P.J., Gasser R.B. (2021). SARS-CoV-2 seroprevalence worldwide: A systematic review and meta-analysis. Clin. Microbiol. Infect..

[120] Böger B., Fachi M.M., Vilhena R.O., Cobre A.F., Tonin F.S., Pontarolo R. (2021). Systematic review with meta-analysis of the accuracy of diagnostic tests for COVID-19. Am. J. Infect. Control..

[121] Gao Z., Xu Y., Sun C., Wang X., Guo Y., Qiu S., Ma K. (2021). A systematic review of asymptomatic infections with COVID-19. J. Microbiol. Immunol. Infect..

[122] Lucan S.C., Goodwin S.K., Lozano M., Pak S., Freitas M. (2021). Severe acute respiratory syndrome coronavirus 2 (SARS-CoV-2) testing for essential food production workers: Evolving thinking, pilot testing, and lessons learned. Public Health.

